# Effects of Internet-Based Cognitive Behavioral Therapy for Harmful Alcohol Use and Alcohol Dependence as Self-help or With Therapist Guidance: Three-Armed Randomized Trial

**DOI:** 10.2196/29666

**Published:** 2021-11-24

**Authors:** Magnus Johansson, Anne H Berman, Kristina Sinadinovic, Philip Lindner, Ulric Hermansson, Sven Andréasson

**Affiliations:** 1 Department of Global Public Health Karolinska Institutet Stockholm Sweden; 2 Department of Clinical Neuroscience Centre for Psychiatry Research Karolinska Institutet Stockholm Sweden; 3 Department of Psychology Uppsala University Uppsala Sweden

**Keywords:** alcohol dependence, alcohol use disorders, internet-based interventions, internet-based cognitive behavioral therapy, ICBT, cognitive behavioral therapy, CBT, eHealth, alcohol use, substance abuse, outcomes, help-seeking behavior, internet-based interventions, alcohol dependence, mobile phone

## Abstract

**Background:**

Alcohol use is a major contributor to health loss. Many persons with harmful use or alcohol dependence do not obtain treatment because of limited availability or stigma. They may use internet-based interventions as an alternative way of obtaining support. Internet-based interventions have previously been shown to be effective in reducing alcohol consumption in studies that included hazardous use; however, few studies have been conducted with a specific focus on harmful use or alcohol dependence. The importance of therapist guidance in internet-based cognitive behavioral therapy (ICBT) programs is still unclear.

**Objective:**

This trial aims to investigate the effects of a web-based alcohol program with or without therapist guidance among anonymous adult help-seekers.

**Methods:**

A three-armed randomized controlled trial was conducted to compare therapist-guided ICBT and self-help ICBT with an information-only control condition. Swedish-speaking adult internet users with alcohol dependence (3 or more *International Classification of Diseases*, *Tenth Revision* criteria) or harmful alcohol use (alcohol use disorder identification test>15) were included in the study. Participants in the therapist-guided ICBT and self-help ICBT groups had 12-week access to a program consisting of 5 main modules, as well as a drinking calendar with automatic feedback. Guidance was given by experienced therapists trained in motivational interviewing. The primary outcome measure was weekly alcohol consumption in standard drinks (12 g of ethanol). Secondary outcomes were alcohol-related problems measured using the total alcohol use disorder identification test-score, diagnostic criteria for alcohol dependence and alcohol use disorder, depression, anxiety, health, readiness to change, and access to other treatments or support. Follow-up was conducted 3 (posttreatment) and 6 months after recruitment.

**Results:**

During the recruitment period, from March 2015 to March 2017, 1169 participants were included. Participants had a mean age of 45 (SD 13) years, and 56.72% (663/1169) were women. At the 3-month follow-up, the therapist-guided ICBT and control groups differed significantly in weekly alcohol consumption (−3.84, 95% Cl −6.53 to −1.16; t_417_=2.81; *P*=.005; Cohen *d*=0.27). No significant differences were found in weekly alcohol consumption between the self-help ICBT group and the therapist-guided ICBT at 3 months, between the self-help ICBT and the control group at 3 months, or between any of the groups at the 6-month follow-up. A limitation of the study was the large number of participants who were completely lost to follow-up (477/1169, 40.8%).

**Conclusions:**

In this study, a therapist-guided ICBT program was not found to be more effective than the same program in a self-help ICBT version for reducing alcohol consumption or other alcohol-related outcomes. In the short run, therapist-guided ICBT was more effective than information. Only some internet help-seekers may need a multisession program and therapist guidance to change their drinking when they use internet-based interventions.

**Trial Registration:**

ClinicalTrials.gov NCT02377726; https://clinicaltrials.gov/ct2/show/NCT02377726

## Introduction

### Background

Alcohol consumption causes substantial health loss. It is the leading risk factor for both mortality and disability-adjusted life years worldwide among those aged 15-49 years, with 3.8% of female deaths and 12.2% of male deaths attributable to alcohol use [1]. There are dose-response relationships between alcohol consumption and many major diseases [2]. Heavy use over time, as discussed by Rehm et al [3], is responsible for most of the attributable burden of disease, mortality, and social consequences of alcohol. When alcohol use is diagnosed as alcohol dependence (*International Classification of Diseases*, *Tenth Revision* [*ICD-10*]), it is characterized by impaired control and continued heavy use despite negative consequences [4]. The prevalence of alcohol dependence is estimated to be 2.6% worldwide and 3.7% in Europe [5]. In Sweden, 4% of adults are estimated to fulfill the diagnostic criteria for alcohol dependence [6].

Different interventions are available to reduce an individual’s alcohol consumption. Brief interventions, often used in primary care settings, are effective in reducing alcohol consumption [7]. Psychological and pharmacological treatments have also shown effects in terms of reduced alcohol consumption (eg, cognitive behavioral therapy [CBT] vs minimal intervention, g=0.67 [8]) and in terms of abstinence from alcohol (eg, acamprosate vs placebo, g=0.36 [9]). However, there have been problems with the implementation of brief interventions; that is, health professionals have limited time or might be reluctant to address alcohol use [10]. Only approximately 7% of people with substance use disorders are estimated to receive at least minimal treatment [11]. Possible reasons for not seeking treatment are lack of access, shame, stigma, not wanting to change one’s alcohol use, or a wish to deal with it by oneself [12,13].

Internet-based interventions can help overcome some of the problems associated with implementation, limited accessibility, and stigma [14]. The internet has developed over the past decades from being an alternative way of finding health-related information to being the common way. Approximately >85% of Swedes use the internet to find information about health or medicine, and approximately 40% do so at least every month [15]. Internet-based interventions for reducing alcohol use have been developed during the past 20 years [16] and include content similar to that of face-to-face interventions, such as personal normative feedback, motivational interviewing (MI), and CBT, intended to motivate the user to reduce their drinking and give them strategies to do so [17]. In a Cochrane review of digital alcohol interventions, including internet-based interventions (37/57, 65% studies), the effect compared with no or minimal interventions was 23 g (95% CI 15 to 30) of less alcohol consumed weekly. According to a recent individual patient data meta-analysis of internet-based alcohol interventions, the effect on alcohol consumption compared with various controls was −22 g less per week (95% CI −8.7 to −34.6) [18]. The same meta-analysis also found that guided internet-based alcohol interventions (with human guidance from health professionals or trained volunteers) are more effective than unguided (fully automated) interventions (−67.8 g alcohol per week, 95% CI −121.1 to −14.5).

However, most of the previous studies on digital and internet-based alcohol interventions have been on brief interventions, such as personal normative feedback, and have been limited to at-risk populations, such as students [17,19]. More extended internet-based alcohol interventions are intended to be used over a number of weeks or sessions and are usually internet CBT (ICBT), for example, based on treatments for alcohol dependence, such as relapse prevention or behavioral self-control training, often combined with principles from MI [19,20]. There are indications that longer, multisession interventions are more effective than shorter or single-session internet-based alcohol interventions [21]. A literature search of multisession internet-based alcohol interventions revealed 14 randomized controlled trials of ICBT aimed at drinkers among the general public with at least hazardous use [22-35] (see Multimedia Appendix 1 for further details). In 5 of these studies [23,25,27,28,35], ICBT as self-help was significantly more effective in reducing alcohol consumption than minimal control interventions or waiting lists. However, several previous large studies did not find a significant difference between self-help ICBT and minimal control [26,32,34]. Therapist-guided ICBT for alcohol was tested in 5 of the 14 previous studies and was found to be more effective than waiting list in 4 studies [22,27,28,35] and unguided self-help in 2 studies [24,28]. However, therapist-guided interventions were not more effective than self-help interventions in the 2 most recent studies on ICBT for alcohol [27,35]. Although many of the participants in previous studies of ICBT programs for alcohol have had alcohol use disorder identification test (AUDIT)–scores indicating a high level of alcohol-related problems, there is a need for studies on internet-based interventions that are aimed specifically at people with harmful use or alcohol dependence [19].

As described above, the effects of ICBT programs, as well as therapist-guided ICBT for alcohol, are still unclear. This could be explained by the fact that many trials have used small sample sizes and included users with different levels of problems (eg, included risky alcohol users), who may change their drinking more easily or to a lesser extent. Most previous studies have also used waiting list control conditions or open (unblinded) design, possibly making the control groups disappointed or less likely to change [36]. In this study, the sample size was larger than in previous studies that investigated the effect of guided ICBT. The participants were also blinded to the interventions that the other participants received. The purpose of this randomized controlled trial was to investigate the effects of a web-based alcohol program with or without therapist guidance among anonymous adult help-seekers with harmful use or alcohol dependence.

### Hypotheses

The hypotheses of the trial were:

1. A therapist-guided ICBT program would lead to a greater reduction in alcohol consumption and alcohol-related problems than information alone.
2. A self-help ICBT program would lead to a greater reduction in alcohol consumption and alcohol-related problems than information alone.
3. A therapist-guided ICBT program would lead to a greater reduction in alcohol consumption and alcohol-related problems than a self-help ICBT program.

## Methods

### Study Design

In a three-arm randomized controlled trial with a parallel design, participants were randomly assigned to an internet-delivered CBT (ICBT) program as self-help, with therapist guidance or information control in a ratio of 1:1:1 and a block size of 30. The trial was approved by the Stockholm Regional Ethical Review Board (No. 2014/1758-31/2).

### Recruitment

#### Overview

Participants were recruited at the Swedish internet site Alkoholhjälpen [37], an open access website that provides information and a discussion forum for individuals seeking web-based help for alcohol consumption. The site has been publicly accessible since 2007. During the recruitment period of this study, Alkoholhjälpen had approximately 20,000 unique visitors every month and approximately 100 new forum posts every day. All service use was free of charge, and no advertising was allowed on the website. All visitors on Alkoholhjälpen from March 2015 to March 2017 were invited to participate in a study to develop and test different forms of internet-delivered support for changing alcohol habits. Interested users were informed that they would answer a survey and be randomized to one of three different forms of support but were not informed about the specifics. Adult individuals who gave their informed consent were instructed to create a personal account with a unique username and password. They were then directed to a screening page where they were required to give informed consent for participation in the study, answer demographic questions, questions in AUDIT [38], and questions about alcohol dependence (*ICD-10*) criteria.

#### Inclusion Criteria

Individuals were included if they had harmful use (defined as >15 total score in AUDIT) or alcohol dependence (defined as 3 or more *ICD-10* criteria). Registrants who did not meet either of these criteria were informed that they did not qualify for the study and were invited to use the open parts of the website. To be able to complete the registration, the participants needed to understand written Swedish and be computer literate enough to access and navigate the website via a computer, tablet, or smartphone. Before registering, potential participants were also informed that the interventions were not intended for users who were experiencing withdrawal symptoms, psychosis, schizophrenia, bipolar disorder, or suicidal thoughts.

#### Baseline Assessment

Eligible participants were asked to complete web-based baseline questionnaires, including primary and secondary measures (*Measures* section). All participants were treated as anonymous users. They were asked to provide an email address and a mobile phone number for notifications and follow-up reminders. Email and phone numbers were neither verified nor used for identification or for any other purposes. Participants whose assessments showed indications of an increased risk of suicide (Montgomery Asberg Depression Rating Scale–Self-rated [MADRS-S] item 9>3) at baseline or follow-up received a message offering additional support with phone numbers to call in case of emergency. No eligible participants were excluded based on baseline assessment. The baseline questionnaires were followed by a survey on why participants chose to use web-based services and their preferences regarding such services. Data from this survey will be presented elsewhere.

#### Randomization

Participants who completed the baseline measures were randomized according to a fully automated and concealed procedure on the web-based platform. Participants were assigned to one of 3 groups: (1) self-help ICBT: a self-help program; (2) therapist-guided ICBT: a program with web-based guidance from a therapist; or (3) control: information on changing alcohol habits. Participants were blinded to the kind of support received by participants in the other groups.

### Intervention Groups

#### Self-help ICBT

**Figure 1:**
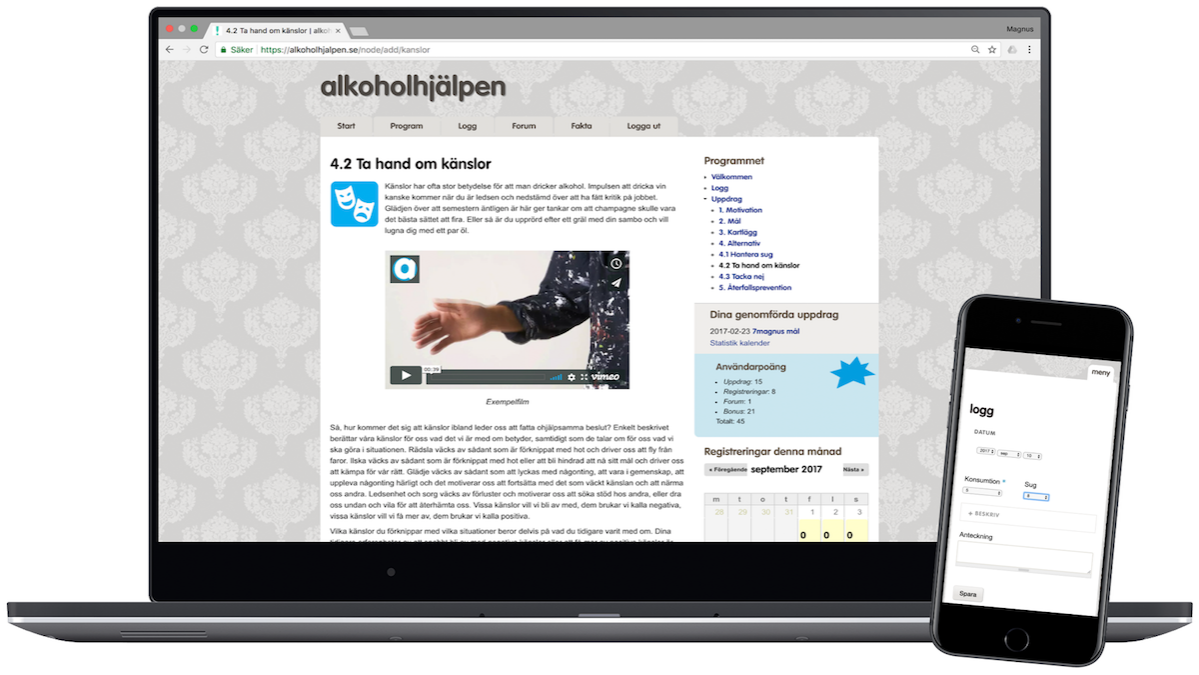
Program module on a computer and the drinking calendar on a smartphone.

Directly following randomization, the self-help ICBT group was given access to the program. The program was based on self-help materials used in previous studies on the internet and in specialist care [24,34,39,40]. Content and exercises in the program were based on MI [41,42], relapse prevention [43,44], and behavioral self-control [45,46]. The program was divided into 5 main modules, 3 extra problem-solving modules, and 10 fact sheets (refer list of modules in Table 1). The length of the program was approximately 17,000 words in total, with 5500 words in the extra modules and 3000 words in the fact sheets. The module texts were alternated with checklists and open questions that prompted the user to give their view of the content in relation to their own situation. The modules also included videos with examples or expert-interviews. Refer to Figure 1 for example pictures of the program. Automatic reminders with suggestions on what module to work on were sent at 1-4, 6, and 8 weeks. Users were also encouraged to register alcohol consumption or craving, as well as details of the situation when they drank or experienced a craving. This was done in a private drinking calendar included in the web program, which could be used daily or for a whole week retrospectively. Continual feedback on the users’ alcohol consumption was offered through a private statistics page. Here, users could see their average personal consumption in standard drinks weekly, monthly, and in total, as well as the number of days drinking, the number of days sober, and binge drinking occasions. In addition, they could also view a summary of their own risk situations. To allow participants to complete the modules at the recommended pace, with room for some delay, the participants had access to the program for 12 weeks after allocation.

**Table 1:** Program modules and number and percentage of participants in the therapist-guided and self-help internet-based cognitive behavioral therapy (ICBT) who used each module.

Module^a^		Therapist-guided ICBT (n=386), n (%)	Self-help ICBT (n=391), n (%)
Motivation (including brief feedback on assessment)		272 (70.5)	265 (67.6)
Drinking-goal and self-control		202 (52.3)	172 (43.8)
Behavioral analysis of drinking and risk situations		148 (38.3)	113 (28.8)
**General problem solving**		102 (26.4)	73 (18.6)
	Handling cravings	103 (26.7)	72 (18.4)
	Handling feelings	77 (19.9)	46 (11.7)
	Drink-refusal skills	65 (16.8)	35 (8.9)
Preventing relapse		63 (16.3)	34 (8.6)

^a^All modules were available to the user from the start. Each module contained general information, audio or video, examples, and exercises. Additional fact sheets included in the program concerned blood alcohol level, anxiety, depression, anger, stress, managing thoughts, relaxation, sleep, leisure activities, and communication.

#### Therapist-Guided ICBT

Participants in the therapist-guided ICBT group had the same access to the same program as the self-help ICBT described above but the drinking calendar and module answers were shared with a therapist. The therapist-guided ICBT group could communicate with the therapist through asynchronous SMS text messages on the intervention website during the 12 weeks of the program. The guidance from the therapist focused on motivating the user to continue using the program and change their drinking. Each time the participant had completed any of the modules, the therapist wrote personal feedback and answered any questions about the program via private comments on the web platform. The feedback highlighted parts of what the participant had stated in the exercises included in each module, which were important from an MI or CBT perspective. Users who did not use or stopped using the program for several weeks were reminded by the therapist 2 times through personal messages on the website (with notification on mail). The 3 therapists involved had several years of experience on the Swedish alcohol helpline [47] and had all reached an approved level in phone-based MI before entering the study. They had been trained in ICBT and received regular supervision from the first author, who is a trained therapist with several years of experience with CBT and ICBT programs.

#### Control

The control group was given access to text-only information on changing their alcohol habits based on the text Alcohol and you [41]. The information material was equivalent to 5 pages of printed text.

All 3 groups also had access to the discussion forum on the website as well as facts on alcohol and health and information about how to find additional support within the health care or social welfare system. Communication between the server hosting the intervention and the participant was encrypted and protected with an individual login name and password.

### Measures

#### Overview

Follow-up was conducted 3 (after treatment), 6, 12, and 24 months after recruitment. In this paper, the results from the 3- and 6-month follow-ups are presented. Primary and secondary outcomes were assessed at all time points.

#### Primary Outcome

The primary outcome was the difference between the groups in alcohol consumption and mean weekly standard drinks. The number of standard drinks each of the 7 days in the preceding week was self-reported using the timeline follow-back (TLFB) method [48]. One standard drink contains 12 g of pure alcohol, according to the Swedish definition. The TLFB has been shown to be a valid and reliable procedure to document recent drinking when administered via the internet [49] and in a 7-day version [50].

#### Secondary Outcomes

Alcohol-related outcomes were assessed with a number of different instruments. AUDIT [38] is a 10-item instrument that covers both alcohol consumption and problems and has been validated in Swedish and via the computer [51,52]. The total AUDIT score was used as a continuous measure of alcohol-related problems. For description at baseline as well as for assessment of nonimproved and deteriorated participants at the 6-month follow-up, the AUDIT score was categorized into 4 zones as follows: I nonproblematic, 0-6p; II hazardous use, 7-15p; III harmful use, 16-19p; and IV probable dependence, 20-40p. The sum of the 3 first items (AUDIT-C) was also used to assess alcohol consumption [53]. Alcohol dependence was assessed by the number of self-reported alcohol dependence criteria during the past year according to *ICD-10* [4]. Alcohol use disorder was assessed by the number of self-rated alcohol use disorder criteria during the past year according to the *Diagnostic and Statistical Manual of Mental Disorders* (Fifth Edition) (*DSM-5*) [54]. Measures of both alcohol dependence and alcohol use disorder diagnostic criteria were included to facilitate comparisons between the study population and diverse community and clinical populations reported in the research literature, where problem severity level can be assessed according to both *ICD-10* and *DSM-5*.

At the 3- and 6-month follow-ups, the time frame for AUDIT, *ICD-10*, and *DSM-5* was changed from 12 months to 3 months. The number of nondrinking days, number of binge drinking days (defined as ≥3 drinks for women and ≥4 drinks for men), the average number of drinks on drinking days, and low-risk consumption at follow-up (≤14 drinks per week for men and ≤9 drinks per week for women and no binge drinking) were also assessed using the TLFB. Health-related quality of life was assessed using the EuroQol-5 dimensions (EQ-5D-5L). An index score was calculated with Crosswalk value sets, using the United Kingdom as a reference [55,56]. Symptoms of depression were measured using the total score of MADRS-S [57,58]. Symptoms of anxiety were measured using the total score of the Generalized Anxiety Disorder Assessment-7 items (GAD-7) [59,60]. *ICD-10*, *DSM-5*, MADRS-S, and GAD-7-scores were categorized for description at baseline (see Multimedia Appendix 2 for details). Use of other support was assessed by 4 questions covering who and where participants talked to someone about their alcohol problems and which medication or which other internet resources they had used regarding alcohol.

#### Additional Measures

Readiness to change was measured at all time points with 2 visual analog scales [61], where users responded on a scale of 0-10 from “I am not ready to reduce/quit my drinking” (0) to “I am very much ready to reduce/quit my drinking” (10). Working alliance was measured 3 and 6 weeks after randomization when all participants were invited via one email and message to log in to the website and answer the Session Rating Scale [62] regarding the use of the website and the intervention that they had received. After each module in the program, users in the therapist-guided ICBT and self-help ICBT groups could rate, on a scale of 0 to 5 stars, how helpful they found the module to be. All uses of the intervention were logged for each user. Participants who completed 4 or more modules in the program were regarded as treatment completers.

### Follow-up

Follow-up was conducted at 3 and 6 months after recruitment. Based on previous web-based studies, high attrition from follow-up could be expected [63]. Reminders and incentives [64] were used to prevent attrition without affecting the intended target group by forcing participants to have personal contact or identify themselves. At follow-up, participants were emailed a link or redirected when logging in on the intervention website to the follow-up questionnaires. The email included information that all participants who completed follow-ups would have a 0.8% (1/120) chance of receiving a free iPad. The same questionnaires, with all primary and secondary outcomes used at baseline, were also used at follow-up and adjusted for the time since the last assessment (3 months). Participants who did not respond to this initial request received up to 5 automated email reminders, a manual email reminder, and a mobile SMS text message. Additional follow-ups at 12 and 24 months after recruitment have recently been completed but have not yet been analyzed.

### Sample Size

Sample size was determined a priori using an effect-size estimate. We aimed to detect a Cohen *d*=0.2 in 2-group comparisons using 2-tailed *t* tests at follow-ups, which based on SDs from a study of alcohol treatment in primary care [40], equated to a between-group difference of Δmean=3.7 drinks. With α=.05 and 80% power, n=394 per group was required for the desired effect size, totaling n=1182. However, to allow analyses of observed data only, assuming 50% missing data at follow-up, the enrollment goal was increased to n=2400.

### Analytic Plan and Statistical Procedure

All statistical analyses were two-sided tests and, unless otherwise specified, used a significance level of *P*<.05. Factorial analysis of variances was used to test differences in baseline measures between users who were retained and those who were lost to follow-up, including interactions between groups and lost to follow-up (at either 3 months or 6 months). Differences in categorical measures were analyzed using the chi-square test. All tests were performed using SPSS version 25 (IBM Corporation).

In accordance with the original protocol, differences in observed means at each follow-up were analyzed with *t* tests under the “missing at random” assumption; however, significant contrasts were supplemented with tipping point sensitivity analyses that systematically imputed missing data at a group level across a range of plausible mean values in the 2 nonrespondent groups (with the same SD) [65,66]. A custom R function was developed that, per contrast, created a matrix of all possible combinations of group-level imputed means (in steps of 0.1 and within the plausible range) of the 2 nonrespondent groups, calculated the new whole-group means and SDs, performed a *t* test using this summary data, and saved the *P* value. This allowed us to estimate the conditions under which (means among nonrespondents) the contrast would no longer be significant (tipping point), thereby testing the appropriateness of the “missing at random” assumption. Two-group *t* tests were corrected for multiple comparisons by considering *P*<.02 as significant, corresponding to the Bonferroni adjustment.

Before analyzing the data, the decision was made to supplement the original analytic protocol with mixed-effects modeling that would be fully compliant with the intention-to-treat principle and better equipped to handle the presumed high degree of missing data [63]. By modeling data at both group (fixed) and individual (random) levels, mixed models are well-suited for data from repeated observations (modeling clustering of data at an individual level) [67], and maximum likelihood estimation is used to handle missing data [68]. Analyzing outcomes with (generalized) mixed-effects models also allowed the use of family functions that were more appropriate for the distribution of the outcome. Weekly alcohol consumption (as well as other outcomes based on TLFB) was analyzed using generalized linear mixed models [69] with a negative binomial distribution and log link to avoid overestimation of effects [70]. AUDIT, *ICD-10*, *DSM-5*, GAD-7, MADRS-S, readiness, and EQ-5D-5L were analyzed with linear mixed models. Mixed-effects modeling was performed using SPSS 25. First, a random intercept model was specified to calculate an interclass correlation score [71]. After visually inspecting the average and individual growth curves, an unconditional model was specified by adding both linear and quadratic time (time^2^) as predictors. A conditional growth model was then specified by adding the self-help ICBT and therapist-guided ICBT groups as dummy-coded variables to be compared against the control (reference), together with the time×group and time^2^×group interactions. Different covariance structures for random effects and errors were tested, and a likelihood ratio test was used to assess which model best fitted the data [67]. There was a significant dependency among the observations (intraclass correlation=0.62) as well as significant individual variability in the initial level (intercept *P*<.001) and rate of change (slope *P*<.001) in the primary outcome.

## Results

### Participants

**Figure 2:**
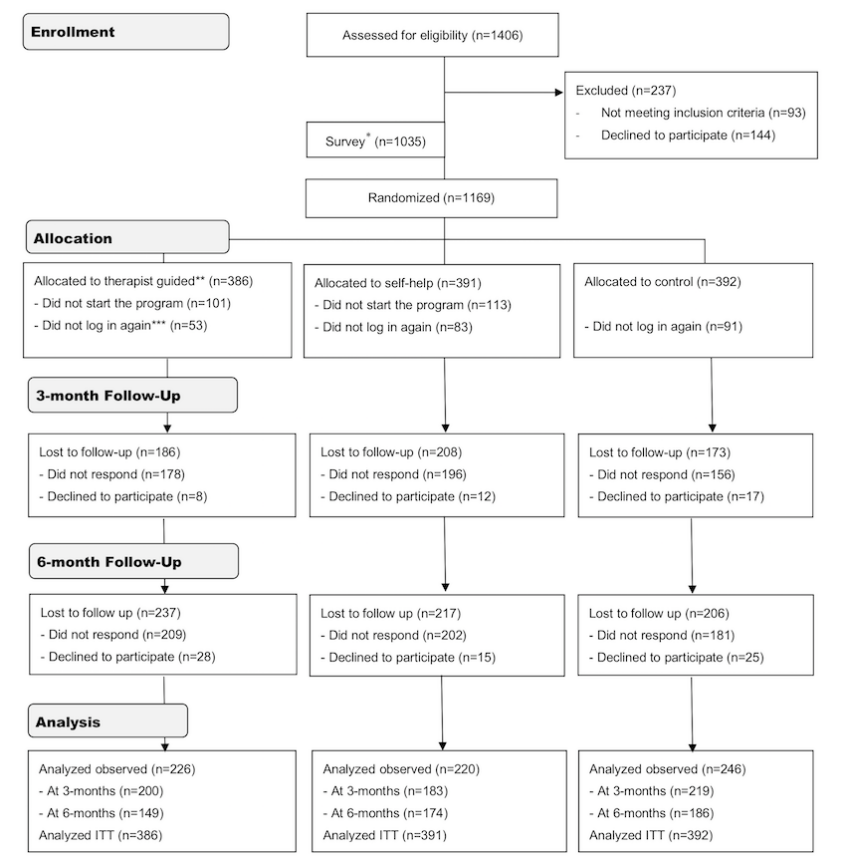
CONSORT (Consolidated Standards of Reporting Trials) flow diagram. *All included participants were asked to complete a survey on why they had chosen to use web-based services and their preferences regarding such services. **Overall, 390 users were randomized to the therapist group, but 4 of them (due to a temporary technical error) never completed the baseline assessment and were never allocated to the intervention. ***Users who did not log in a second time after allocation to intervention. ICBT: internet-based cognitive behavioral therapy; ITT: intention-to-treat.

A total of 1169 participants were randomly allocated to the 3 study arms (refer to Figure 2 for the flow chart). This was lower than the target sample size required to adjust analyses on observed data only for estimated attrition at follow-up but only negligibly smaller than in the raw power calculation with estimated missing data (n=1182). Nonetheless, recruitment ceased after the prespecified 24 months recruitment window for funding reasons. Individuals who declined participation after the screening did not differ from those included in the AUDIT (mean 22.1, SD 5.6 vs mean 22.6, SD 6.5; t_1308_=1.00; *P*=.32) or *ICD-10* (mean 4.2, SD 1.3 vs mean 4.2, SD 1.2; t_1308_=0.10; *P*=.92) scores. The randomized participants had a mean age of 45 years (SD 13), and 56.72% (663/1169) were women. During the past year 5.05% (59/1169) had talked to someone in specialized care and 18.99% (222/1169) to a professional about their alcohol use. Participants indicated significantly higher mean readiness to reduce their drinking compared with mean readiness to quit drinking (mean 8.8, SD 1.9 vs mean 5.7, SD 3.6; t_1168_=32.4; *P*<.001). The full demographic and clinical variables at baseline are shown in Multimedia Appendix 2.

### Loss to Follow-up

Attrition was 49% at the 3-month and 66% at the 6-month follow-up. The number of participants who completed at least one of the follow-ups at 3 or 6 months was 692 (59.2%). There were no significant differences in the number of participants in each group who completed at least one of the follow-ups (*Χ*^2^_2_=3.5; *P*=.17). Comparisons between those lost to follow-up and those who completed at least one follow-up showed greater baseline drinking, as well as greater severity on most baseline measures, among those who were lost to follow-up (Table 2). However, no significant interactions between group assignment and being lost to follow-up were found for baseline outcome variables.

**Table 2:** Differences in baseline variables between participants who were lost to follow-up (both assessment) and those who completed at least one assessment (N=1169).

Characteristics	Therapist-guided ICBT^a^ (n=386), mean (SD)			Self-help ICBT (n=391), mean (SD)			Control (n=392), mean (SD)			Comparison			
	Retained (n=226)	Lost (n=160)	Retained (n=220)		Lost (n=171)	Retained (n=246)		Lost (n=146)	Lost vs retained			Group×lost	
									*F* test (*df*)		*P* value	*F* test (*df*)	*P* value
Weekly drinks	23.7 (16.7)	25.9 (16.1)	24.4 (17.5)		28.3 (17.4)	24.4 (17.5)		28.3 (17.4)	11.99 (1)		<.001	0.43 (2)	.65
AUDIT^b^	21.6 (5.4)	22.9 (5.9)	21.9 (5.6)		22.6 (5.5)	21.9 (5.6)		22.6 (5.5)	6.89 (1)		.009	0.42 (2)	.66
AUDIT-C^c^	8.1 (1.8)	8.6 (1.5)	8.1 (1.8)		8.5 (1.7)	8.1 (1.8)		8.5 (1.7)	15.07 (1)		<.001	0.04 (2)	.96
*ICD-10* ^d^	4.2 (1.4)	4.3 (1.3)	4.15 (1.4)		4.5 (1.3)	4.2 (1.4)		4.5 (1.3)	4.39 (1)		.04	1.36 (2)	.26
*DSM-5* ^e^	7.0 (2.4)	7.3 (2.3)	7.07 (2.3)		7.5 (2.1)	7.1 (2.3)		7.5 (2.1)	5.48 (1)		.02	0.14 (2)	.87
MADRS-S^f^	18.9 (8.7)	19.1 (10.0)	17.3 (8.9)		18.8 (8.8)	17.3 (8.9)		18.8 (8.8)	3.87 (1)		.049	0.62 (2)	.54
GAD-7^g^	8.3 (5.3)	9.4 (5.7)	7.7 (5.4)		8.8 (5.2)	7.7 (5.4)		8.8 (5.2)	12.82 (1)		<.001	0.01 (2)	.99
EQ-5D^h^	0.72 (0.19)	0.69 (0.24)	0.73 (0.19)		0.72 (0.18)	0.73 (0.19)		0.72 (0.18)	7.37 (1)		.007	1.05 (2)	.35
Nondrinking days	2.9 (1.97)	2.7 (2.08)	2.85 (2.16)		2.6 (2.08)	2.85 (2.16)		2.6 (2.08)	3.61 (1)		.06	0.01 (2)	.99
Binge drinking days	2.7 (1.9)	3.0 (1.8)	2.8 (2.1)		3.3 (2.1)	2.8 (2.1)		3.3 (2.1)	10.59 (1)		<.001	0.30 (2)	.74
Drinks per drinking day	5.1 (3.7)	5.2 (4.1)	4.8 (3.8)		4.9 (4.2)	4.8 (3.8)		4.9 (4.2)	2.43 (1)		.12	0.86 (2)	.42

^a^ICBT: internet-based cognitive behavioral therapy.

^b^AUDIT: alcohol use disorder identification test.

^c^AUDIT-C: alcohol use disorder identification test consumption questions.

^d^*ICD-10*: *International Classification of Diseases, Tenth Revision*.

^e^*DSM-5*: *Diagnostic and Statistical Manual of Mental Disorders* (Fifth Edition).

^f^MADRS-S: Montgomery Asberg Depression Rating Scale–Self-rated.

^g^GAD-7: Generalized Anxiety Disorder Assessment–7 items.

^h^EQ-5D: EuroQol-5 dimensions.

### Differences in Observed Means

#### Outcomes at 3 Months (Posttreatment)

**Figure 3:**
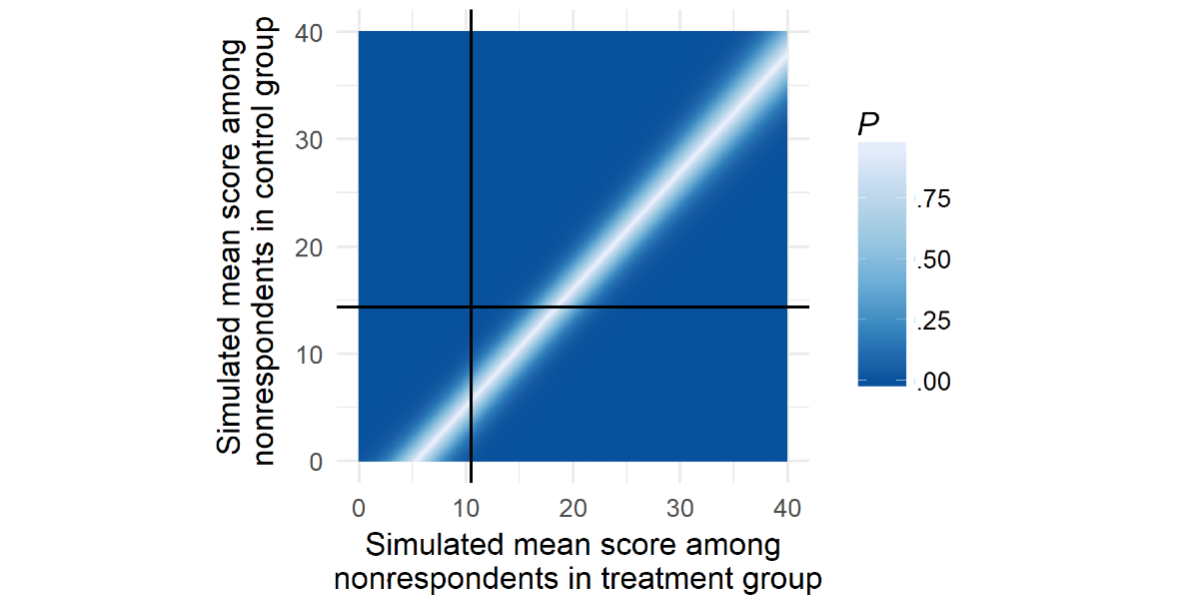
Tipping point analysis of significant contrast in primary outcome at 3 months.

The therapist-guided ICBT group had significantly lower weekly alcohol consumption than the control group at 3 months (mean difference −3.84, 95% Cl −6.53 to −1.16; Cohen *d*=0.27). A tipping point sensitivity analysis revealed that missing data in the control group would need to have a mean of 11.0 to render this contrast insignificant (assuming missing at random in the therapist-guided ICBT group), or that the missing data in the therapist-guided ICBT group would need to have a mean of 13.6 (assuming missing at random in the control group). Refer to Figure 3 for the *P* value heat map for each of the possible combinations of imputed means among nonrespondents in the 2 groups. No significant differences in weekly alcohol consumption were found between the self-help ICBT group and the control group (mean difference −2.41, 95% Cl −5.53 to 0.71) or between the therapist-guided ICBT group and the self-help ICBT group (mean difference −1.43, 95 CI −4.26 to 1.40).

At 3 months (posttreatment) there were significant differences between the therapist-guided ICBT group and the control group in the secondary outcomes AUDIT (mean difference −2.91, 95% Cl −4.33 to −1.50; Cohen *d*=0.39), AUDIT-C (mean difference −0.77, 95% CI −1.34 to −.20; Cohen *d*=0.26), *DSM-5* (mean difference −0.76, 95% CI −1.34 to −0.17; Cohen *d*=0.25), and *ICD-10* (mean difference −0.47, 95% Cl −0.82 to −0.14; Cohen *d*=0.26). A difference was also found between the self-help ICBT and control groups on the AUDIT (mean difference −1.95, 95% Cl −3.44 to −46; Cohen *d*=0.26) at 3 months. No significant differences in secondary outcomes between the therapist-guided ICBT and self-help ICBT were found at 3 months. See Multimedia Appendix 3 for details and Multimedia Appendix 4 for the tipping point analyses of secondary outcomes. Of the participants who completed the 3-month follow-up, 42.9% (258/602) reported low-risk alcohol consumption (14 or fewer drinks per week for men and 9 or fewer drinks per week for women and no binge drinking) at 3 months, with no significant differences between groups (*Χ*^2^_2_=1.9; *P*=.38).

#### Outcomes at 6 Months

There were no significant differences in weekly alcohol consumption among any of the groups at 6 months. The difference in means between the therapist-guided ICBT and control group was −0.60 (95 CI −3.70 to 2.50), between self-help ICBT and control group −0.45 (95% Cl −3.87 to 2.96), and between the therapist-guided ICBT and the self-help ICBT −0.15 (95% Cl −3.70 to 3.41). No significant differences in secondary outcomes were found among any of the groups at 6 months. Low-risk alcohol consumption was reported by 42.6% (217/509) of participants at 6 months, with no significant differences between the groups (*Χ*^2^_2_=0.2; *P*=.92). Participants reported no adverse events because of the intervention. Among participants followed up at 6 months, 3.3% (17/508) had changed their alcohol use to a more severe category according to AUDIT, and 19.5% (99/508) remained in the highest AUDIT category.

### Post Hoc Intention-To-Treat Mixed Models Analysis

The mixed-model intention-to-treat analysis showed a significantly larger decrease in weekly alcohol consumption over time in the therapist-guided ICBT (time×therapist; *P*=.02) compared with the control but not in the self-help ICBT (time×self-help; *P*=.09) compared with the control (refer models in Table 3). A model comparing only the participants in the therapist-guided ICBT and self-help ICBT did not reveal any significant effect of group×time (*P*=.57). There was a significant decrease in weekly alcohol consumption over time for participants in all 3 groups according to the estimate of time in the unconditional model (*P*<.001).

**Table 3:** Post hoc mixed models of weekly alcohol consumption, alcohol-related problems, alcohol use disorder, and alcohol dependence^a^.

Characteristics	Weekly drinks^b^		AUDIT^c,d^			AUDIT-C^c,e^			*ICD-10* ^c,f^			*DSM-5* ^c,g^	
	B (SE)	*P* value	B (SE)	*P* value	B (SE)		*P* value	B (SE)		*P* value	B (SE)		*P* value
Intercept	3.06 (0.04)	>.001	22.03 (75.44)	>.001	8.29 (82.50)		>.001	4.21 (58.59)		>.001	7.14 (58.38)		>.001
Time	−0.92 (0.14)	>.001	−10.40 (−13.27)	>.001	−4.00 (−13.40)		>.001	−1.42 (−7.35)		>.001	−2.68 (−8.29)		>.001
Time^2^	0.17 (0.07)	.02	3.32 (8.47)	>.001	1.32 (8.84)		>.001	0.47 (4.82)		>.001	0.90 (5.55)		>.001
Therapist-guided ICBT^h^	−0.03 (0.05)	.55	0.07 (0.16)	.87	0.03 (0.20)		.84	0.03 (0.29)		.77	0.03 (0.16)		.87
Self-help ICBT	0.00 (0.05)	.94	0.20 (0.49)	.62	0.01 (0.04)		.97	0.09 (0.86)		.39	0.12 (0.67)		.50
Therapist-guided ICBT×time	−0.48 (0.20)	.02	−4.51 (−3.99)	>.001	−1.40 (−3.26)		.001	−0.63 (−2.27)		.02	−0.98 (−2.10)		.04
Therapist-guided ICBT×time^2^	0.23 (0.11)	.03	1.84 (3.24)	.001	0.62 (2.89)		.004	0.22 (1.57)		.12	0.34 (1.46)		.14
Self-help ICBT×time	−0.35 (0.21)	.09	−3.02 (−2.60)	.009	−0.59 (−1.35)		.18	−0.35 (−1.24)		.22	−0.43 (−0.90)		.37
Self-help ICBT×time^2^	0.17 (0.11)	.13	1.18 (2.01)	.04	0.25 (1.14)		.26	0.10 (0.69)		.49	0.11 (0.44)		.66

^a^Time was coded in 3-month-periods (0, 1 and 2). A model with quadratic time (time^2^) was chosen since it fitted the data better than a model with linear time only. Reference group was control.

^b^Generalized linear mixed-model. Neg-binominal distribution, dispersion coefficient: 0.944. Covariance structure for random effects Variance component and for repeated effects Diagonal.

^c^Linear mixed-model. Covariance structure for random effects Variance component and for repeated effects First-Order Autoregressive.

^d^AUDIT: alcohol use disorder identification test.

^e^AUDIT-C: alcohol use disorder identification test consumption questions.

^f^*ICD-10*: *International Classification of Diseases, Tenth Revision*.

^g^*DSM-5*: *Diagnostic and Statistical Manual of Mental Disorders* (Fifth Edition).

^h^ICBT: internet-based cognitive behavioral therapy.

According to the mixed-model analysis of secondary outcomes, a significantly larger decrease in AUDIT over time was found for both therapist-guided ICBT and self-help ICBT compared with the control group, as shown by therapist-guided ICBT×time (*P*<.001) and self-help ICBT×time (*P*=.03). There were also significant therapist-guided ICBT×time group effects for the AUDIT-C (*P*=.01), *ICD-10* (*P*=.02), and *DSM-5* (*P*=.04) diagnostic criteria. No other significant time×group effects were found in the mixed-model analysis of secondary outcomes. Over time, there was a significant decrease among all participants on the AUDIT, AUDIT-C, *ICD-10*, *DSM-5*, MADRS-S, GAD-7, binge drinking days, and drinks on drinking days as well as a significant increase in EQ-5D-5L and nondrinking days. See Multimedia Appendix 5 for additional models.

Changes at follow-ups showed strong correlations between similar outcome variables, such as alcohol dependence and AUD, GAD7, and MADRS-S or weekly drinking and binge drinking days but only moderate or weak correlations between alcohol-related variables and other outcomes. Refer to Multimedia Appendix 6 for further details.

### Intervention Use and Rating

The number of modules completed by the therapist-guided ICBT (mean 3.3, SD 3.5) was significantly higher (t_775_=2.9; *P*=.004) compared with the self-help ICBT (mean 2.6, SD 3.2); however, there was no significant difference in the number of calendar entries (therapist-guided ICBT: mean 39, SD 61 and self-help ICBT: mean 37; SD 102; t_776_=0.60; *P*=.58). In the therapist-guided ICBT, 39.9% (154/386) were treatment completers, and in the self-help ICBT, 30.4% (119/386) (*Χ*^2^_1_=6.5; *P*=.01). In the therapist-guided ICBT, 58% (224/386) sent at least one message to their therapists. They sent a mean 4.7 (SD 4.7) messages and received a mean 6.0 (SD 4.1) from their therapist. Refer to Table 1 for the details of program use. The number of participants who used the discussion forum was higher in the control group compared with the therapist-guided ICBT group (107/386, 27.7% vs 71/391, 18.2%; *Χ*^2^_1_=8.7; *P*=.003). However, there were no significant differences between self-help ICBT (88/392, 22.4%) and the therapist-guided ICBT or the control group in forum use. Of those who participated in at least one follow-up, 47% (325/692) answered that they had talked to a professional about their alcohol use since entering the study, there were no significant differences between the groups. Participants gave a significantly higher rating of the working alliance with the intervention (Session Rating Scale) in the therapist-guided ICBT (mean 27, SD 11; t_269_; *P*<.001) and self-help ICBT (mean 29, SD 10; 185/392, 47.1%; t_305_; *P*<.001) compared with the information control group (mean 19, SD 12). The mean rating of program modules were 3.8 (SD 1.0) on a scale 0-5, with no difference in rating between the therapist-guided ICBT and the self-help ICBT (t_731_=0.69; *P*=.49).

## Discussion

### Principal Findings

The aim of this web-based randomized controlled trial was to investigate the effect of a program for harmful alcohol use and alcohol dependence, delivered as self-help ICBT or therapist-guided ICBT. The results only partly confirmed the first hypothesis. Participants randomized to therapist-guided ICBT reduced their weekly alcohol consumption as well as alcohol-related problems (measured with AUDIT) and signs of alcohol use disorder significantly more than participants in the control group at the 3-month follow-up. These findings are in line with the results of previous studies on therapist-guided ICBT [22,24,27] but with smaller differences between the groups. The results did not confirm the second hypothesis. Self-help ICBT was not more effective than the control condition in changing alcohol consumption. This is in line with the results of the first study of Alkoholhjälpen [34] and 2 other large studies of publicly available services [26,31]. However, the self-help ICBT group did change their alcohol-related problems significantly more than the controls at 3 months. No support was found for the third hypothesis. There were no significant differences in changed drinking or other outcomes between therapist-guided ICBT and self-help ICBT. This finding differs from our pilot study [24] and the previous study by Blankers et al [28], in which therapist guidance was significantly more effective than self-help, but in line with recent studies by Boß et al [27] and Sundström et al [35].

A possible factor explaining the difference in results between trials could be the intensity of guidance [72]. Participants receiving therapist-guided interventions completed approximately 60% of the programs in the 3 trials for which data are available [22,24,27]. The study by Postel et al [22], showing the largest effects of a therapist-guided intervention, had a high level of guidance, whereas the study by Boß et al [27] had low intensity, with only 33% of the therapist-guided group using the guidance. In this study, 58% (224/386) of patients in the therapist-guided group used the guidance, which was neither low nor high in intensity compared with the previous studies. The differences between the groups were smaller than those in previous studies. This might be explained by the fact that the participants in this trial were blinded to group allocation, which reduced the risk of being negatively affected by being put in a control group [36,73]. The control group in this study reduced their alcohol consumption by 11 weekly drinks between baseline and the 3-month follow-up compared with, for example, 3 drinks for the waiting list in the study by Postel et al [22]. The difference between groups at 3 months faded at 6 months in this trial. The therapist guidance and program ended after 12 weeks; offering more extended support might have increased the effects at 6-months.

The results of this study also suggest that the decrease in alcohol consumption and related outcomes might result from factors other than the interventions that affected all participants or occurred before the randomization. A significant decrease in alcohol consumption and alcohol-related problems occurred in all 3 study groups in the first 3 months, and this decrease remained stable up to 6 months after inclusion. All study participants were recruited based on their initial harmful use or alcohol dependence. Regression toward the mean [74] could explain some of the decreases in alcohol use and other outcomes in all 3 groups. Participants were recruited from a website about changing alcohol use and could be characterized as help-seekers. Even though they did not know what kind of interventions they would receive, they had already taken steps in the direction of changing their alcohol consumption by signing up to the website. This was also indicated by the high mean readiness to change alcohol consumption (8.8/10). All participants also had to answer a large number of assessment questions about their alcohol, an activity that has been shown to lead to reductions in alcohol consumption [75,76].

All 3 groups in this trial had access to a well-established and moderated discussion forum, which might have affected their alcohol use [77,78]. Significantly more participants in the control group used the forum than in the therapist-guided ICBT group, which might indicate that some participants compensated for the lack of human contact in the intervention by using the forum. Symptoms of depression and anxiety were reduced, and health improved over time in all 3 groups but not more in those who received self-help ICBT or therapist-guided ICBT. This finding differs from findings by Boß et al [27], where a multisession program for risky drinking had a small-size effect on depression, stress, and anxiety at follow-up relative to control.

### Generalizability

The generalizability is likely limited to Swedish-speaking people with harmful alcohol use or alcohol dependence seeking help for their drinking on the internet. This study tried to recruit participants that were as similar as possible to the intended target group of the intervention as used in regular service at Alkoholhjälpen. The trial was conducted in the same setting. Information on needed language skills and the limitations of the interventions in helping those with severe psychiatric problems were provided; however, no other criteria were used to exclude participants who fulfilled the criteria set for harmful use or alcohol dependence. There were no differences in alcohol-related problems (AUDIT) or dependence criteria (*ICD-10*) between those who accepted and those who declined participation. The high attrition rate also limits the generalizability of our results.

### Strengths

This randomized controlled study of therapist-guided ICBT and self-help ICBT is one of the largest among anonymous internet help-seekers to date. The study reached a large number of people with harmful use or alcohol dependence, most of whom had not previously received support from specialized care. The number of dependence criteria met by participants was similar to that in a recent Swedish clinical trial in specialized and primary care [40]. The participants in this study were relatively well-educated, full-time employed individuals with stable living arrangements and with equal representation of men and women. This represents most individuals with alcohol dependence [6], a population that is different from those who usually receive treatment for alcohol use disorders [79,80], but that might be reached with internet-based interventions.

### Limitations

Despite great efforts to remind and reinforce participants to answer follow-up questions, attrition was high. Participants lost to follow-up showed some differences to those retained, a factor that limits the generalizability of the results. However, tipping point analyses and the fact that there were no significant arm×attrition interactions on outcomes suggest that no sampling bias was introduced as a result of the attrition. Attrition could be a consequence of allowing users to be relatively anonymous and having a fast and accessible way of signing up for the study, lowering the threshold for engagement. The high attrition also means that the power to detect effect sizes was smaller than planned. In the between-group comparison of the self-help and therapist-guided arms at the 3-month follow-up, observed sample sizes would have given 80% power to detect an effect size of *d*>0.29, which is to be considered a small difference. However, we cannot rule out that the true difference is smaller than this. Adherence to the program was relatively low, with only 30.4% (119/391) completers in the self-help ICBT and 39.9% (154/386) in the therapist-guided ICBT, which is consistent with previous studies on ICBT (Multimedia Appendix 1). Higher adherence might improve the effects of the internet-based program. Owing to the web-based setting, the participants did not go through a clinical diagnostic interview, and some participants may not have been diagnosed as having alcohol dependence had an interview been included in the study design.

### Future Directions

There is still a need for more studies on multisession internet-based interventions for harmful alcohol use and alcohol dependence, including studies with long-term follow-ups. No differences between the groups were found in number of participants that reported low-risk drinking at follow-ups. Only some internet help-seekers might need ICBT and therapist guidance to change their drinking when they use internet-based interventions. Others who did not improve might have benefited from more intensive support. A model of support-on-demand or accelerated care could be tested in future studies on the internet. One important challenge for future studies is to improve follow-up rates and adherence to interventions without reducing the willingness to use the interventions. Increased demands on users to identify themselves or have contact with a professional might make people who wish to remain anonymous or feel ashamed or stigmatized more reluctant to seek support [81]. Treatment-seeking increases the rates of recovery from alcohol dependence [82], and internet-based interventions seem to be a possible way to reach individuals currently not seeking treatment [83]; however, it is still unclear whether internet-based interventions actually increase treatment-seeking. Research on other psychiatric disorders [84] and on internet-based alcohol interventions so far [17] suggest that therapist-guided internet treatment has effects comparable with those of face-to-face treatment; however, more studies are needed that directly compare these interventions, as a recent study by our group has done [85]. In sparsely populated countries such as Sweden, where some people have to travel far to visit a clinic in person, psychological treatment [86], medical management [87], and after care [88] could, in part, be handled with internet-based interventions. More studies are needed to understand how internet-based interventions can be used effectively to improve treatment for people with alcohol dependence.

### Conclusions

In this study, a therapist-guided ICBT program was not found to be more effective than the same program as a self-help ICBT for reducing alcohol consumption or other alcohol-related outcomes. In the short run, therapist-guided ICBT seems to be more effective than information. Only some internet help-seekers might need a multisession program and therapist guidance to change their drinking when they use internet-based interventions.

## Appendix

Multimedia Appendix 1 Controlled trials of internet cognitive behavioral therapy for alcohol use.

Multimedia Appendix 2 Demographic and clinical characteristics at baseline.

Multimedia Appendix 3 Observed mean, SD, and difference between groups.

Multimedia Appendix 4 Tipping point analyses of secondary outcomes.

Multimedia Appendix 5 Mixed models of secondary outcomes.

Multimedia Appendix 6 Correlations between changes in outcomes at follow-ups.

Multimedia Appendix 7 CONSORT-eHEALTH checklist (V 1.6.1).

## Abbreviations

AUDIT: alcohol use disorder identification test
CBT: cognitive behavioral therapy
*DSM-5*: *Diagnostic and Statistical Manual of Mental Disorders* (Fifth Edition)
EQ-5D-5L: EuroQol-5 dimensions
GAD-7: Generalized Anxiety Disorder Assessment-7 items
ICBT: internet-based cognitive behavioral therapy
*ICD-10*: *International Classification of Diseases, Tenth Revision*
MADRS-S: Montgomery Asberg Depression Rating Scale–Self-rated
MI: motivational interviewing
TLFB: timeline follow-back
