# Supplementary material for: Effects of Internet-Based Cognitive Behavioral Therapy for Harmful Alcohol Use and Alcohol Dependence as Self-help or With Therapist Guidance: Three-Armed Randomized Trial
Source: J Med Internet Res. 2021 Nov 24;23(11):e29666. doi: 10.2196/29666 (PMC8663526; doi:10.2196/29666)
Supplement: Multimedia Appendix 1 [file jmir_v23i11e29666_app1.docx]

|  |  | Inclusion women/men | AUDIT  M(SD) | Drinks/ week  M(SD) | Male | Age | Program use | Modul/ session | n | Primary  outcome | Retention at follow-up | | | Significant difference favoring |
| --- | --- | --- | --- | --- | --- | --- | --- | --- | --- | --- | --- | --- | --- | --- |
| **Selhelp ICBT vs minimal/wait-list** | |  |  |  |  |  |  |  |  |  | 2-3m | 6m | 12m |  |
| Riper | 2008 | ≥14/21 drinks/w or 4/6 on occasion | n/a | 37(18) | 51% | 46 | 45% started | 4 | 261 | drinks/week | n/a | 58% | n/a | ICBT |
| Blankers | 2011 | AUDIT>8 or Drinks/week ≥14 | 20(5) | 37(22) | 50% | 42 | 68% started | 7 | 205 | drinks/week | 71% | 60% | n/a | ICBT |
| Wallace | 2011 | AUDIT-C ≥5 | 19(7) | 30 | 43% | 38 | M=2,3 sessions |  | 2652 | drinks/week | n/a | 48% | 33% | no |
| Brendryen | 2013 | FAST ≥3 | n/a | 20(13) | 67% | 38 | 50% ≥3 sessions | 62 | 244 | drinks/week | 62% | 70% | n/a | no |
| Brief | 2013 | AUDIT 5/8 | 18 | 29 | 87% | 32 | M=3,6 visits | 8 | 600 | DDD | 48% | 40% | n/a | ICBT |
| Sinadinovic | 2014 | AUDIT ≥6/8 | 22 | n/a | 55% | 44 |  | 18 | 422 | audit-c | 38% | 35% | 45% | no |
| Boss | 2017 | AUDIT ≥6/8 or Drinks/week ≥14/21 | n/a | 30(16) | 41% | 47 | 40% ≥3 sessions M= 2,5 modules | 5 | 434 | drinks/week | 78% | 62% | n/a | ICBT |
| Sundström | 2019 | AUDIT ≥14/≥ 16 | 23(5) | 34(17) | 49% | 53 | M=5,9 modules | 9 | 166 | drinks/week | 87% | 78% | n/a | ICBT |
| **Selfhelp ICBT vs other** | |  |  |  |  |  |  |  |  |  |  |  |  |  |
| Hester | 2011 | AUDIT = 8+ | 18 | 34 | 44% | 50 | 42% ≥3 sessions |  | 80 | PDA, DDD | 86% | n/a | n/a | ICBT |
| Cunningham | 2012 | AUDIT-C ≥4 | 22 | 32(18) | 59% | 45 | 72% started | 20 | 425 | drinks/week | n/a | 90% | n/a | ICBT |
| Hester | 2013 |  | 25(8) | n/a | 39% |  | M= 7 logins | 5 | 189 | PDA, DDD | 80% | 69% | n/a | no |
| Sinadinovic | 2014 | AUDIT ≥6/8 | 22 | n/a | 55% | 44 |  | 18 | 423 | audit-c | 38% | 35% | 45% | no |
| Cunningham | 2017 | AUDIT-C ≥4 | 20(8) | - |  | 40 | 22% ≥3 sessions | 20 | 490 | audit-c | n/a | 82% | 83% | no |
| **Guided CBT vs selfhelp** | |  |  |  |  |  |  |  |  |  |  |  |  |  |
| Blankers | 2011 | AUDIT>8 or Drinks/week ≥14 | 20(5) | 37(22) | 50% | 42 | 68% started | 7 | 205 | drinks/week | 71% | 60% | n/a | Guided* |
| Sundström | 2016 | AUDIT ≥ 6/8 | 22(5) | 29(17) | 40% | 42 | M=4 modules | 7 | 80 | drinks/week | 64% | n/a | n/a | Guided |
| Boss | 2017 | AUDIT ≥6/8 or Drinks/week ≥14/21 | n/a | 30(16) | 41% | 47 | 61% ≥3 sessions M=3 modules | 5 | 434 | drinks/week | 78% | 62% | n/a | no |
| Sundström | 2019 | AUDIT ≥14/≥ 16 | 23(5) | 34(17) | 49% | 53 | M= 8,4 modules | 13 | 166 | drinks/week | 87% | 78% | n/a | no |
| **Guided CBT vs minimal/wait-list** | |  |  |  |  |  |  |  |  |  |  |  |  |  |
| Postel | 2010 | Drinks/week  15-67/22-99 | 35 | 13 | 46% | 45 | M=8,3 modules | 12 | 156 | drinks/week | 65% | n/a | n/a | Guided |
| Boss | 2017 | AUDIT ≥6/8 or Drinks/week ≥14/21 | n/a | 30 | 41% | 47 | 61% ≥3 sessions M=3 modules | 5 | 434 | drinks/week | 78% | 62% | n/a | Guided |
| Sundström | 2019 | AUDIT ≥14/≥ 16 | 23(5) | 34 | 49% | 53 | M=8,4 modules | 13 | 166 | drinks/week | 87% | 78% | n/a | Guided |
|  |  |  |  |  |  |  |  |  |  |  |  |  |  |  |
|  |  |  |  |  |  |  | *At 6 months  PDA: percent days abstinent, DDD: mean standard drinks per drinking day | | | | | | | |
