# Supplementary material for: Effects of Internet-Based Cognitive Behavioral Therapy for Harmful Alcohol Use and Alcohol Dependence as Self-help or With Therapist Guidance: Three-Armed Randomized Trial
Source: J Med Internet Res. 2021 Nov 24;23(11):e29666. doi: 10.2196/29666 (PMC8663526; doi:10.2196/29666)
Supplement: Multimedia Appendix 2 [file jmir_v23i11e29666_app2.docx]

Demographic and clinical characteristics at baseline (N=1169).

| Characteristics | | Therapist ICBT^a^ (n=386), n (%) | Self-help ICBT  (n=391), n (%) | Control  (n=392), n (%) | All (n=1169),  n (%) | Chi-square  (*df*) | *P* value |
| --- | --- | --- | --- | --- | --- | --- | --- |
| **Gender** | | | | | | | |
|  | Women | 228 (59.1) | 220 (56.3) | 215 (54.8) | 663 (56.7) | 1.4 (2) | .48 |
|  | Men | 158 (40.9) | 171 (43.7) | 177 (45.2) | 506 (43.3) | 1.4 (2) | .48 |
| **Education** | | | | | | | |
|  | University or college | 207 (53.6) | 199 (50.9) | 202 (51.5) | 608 (52) | 11.8 (8) | .16 |
|  | Upper secondary school  high school or equivalent | 153 (39.6) | 153 (39.1) | 172 (43.9) | 478 (40.9) | 11.8 (8) | .16 |
|  | Primary school or folk school | 21 (5.4) | 31 (7.9) | 15 (3.8) | 67 (5.7) | 11.8 (8) | .16 |
| **Residence** | | | | | | | |
|  | Villa or townhouse | 177 (45.9) | 143 (36.6) | 165 (42.1) | 485 (41.5) | 21.2 (16) | .17 |
|  | Rental apartment | 92 (23.8) | 129 (33) | 102 (26) | 323 (27.6) | 21.2 (16) | .17 |
|  | Condominium | 86 (22.3) | 91 (23.3) | 91 (23.2) | 268 (22.9) | 21.2 (16) | .17 |
| **Living circumstances** | | | | | | | |
|  | With partner and children | 137 (35.5) | 140 (35.8) | 147 (37.5) | 424 (36.3) | 7.2 (12) | .84 |
|  | With partner only | 109 (28.2) | 100 (25.6) | 100 (25.5) | 309 (26.4) | 7.2 (12) | .84 |
|  | Alone | 65 (16.8) | 73 (18.7) | 75 (19.1) | 213 (18.2) | 7.2 (12) | .84 |
|  | With children only | 30 (7.8) | 25 (6.4) | 21 (5.4) | 76 (6.5) | 7.2 (12) | .84 |
| **Civil status** | | | | | | | |
|  | Married | 140 (36.3) | 145 (37.1) | 155 (39.5) | 440 (37.6) | 6.9 (8) | .55 |
|  | Cohabiting | 97 (25.1) | 98 (25.1) | 104 (26.5) | 299 (25.6) | 6.9 (8) | .55 |
|  | Single | 101 (26.2) | 97 (24.8) | 95 (24.2) | 293 (25.1) | 6.9 (8) | .55 |
| **Source of income** | | | | | | | |
|  | Employment | 304 (78.8) | 308 (78.8) | 312 (79.6) | 924 (79) | 8.6 (14) | .86 |
|  | Study allowance | 18 (4.7) | 23 (5.9) | 26 (6.6) | 67 (5.7) | 8.6 (14) | .86 |
|  | Pension | 18 (4.7) | 19 (4.9) | 23 (5.9) | 60 (5.1) | 8.6 (14) | .86 |
| **Country of birth** | | | | | | | |
|  | Sweden | 359 (93.0) | 364 (93.1) | 362 (92.3) | 1085 (92.8) | 9.3 (10) | .50 |
|  | Other Nordic country | 12 (3.1) | 13 (3.3) | 18 (4.6) | 43 (3.7) | 9.3 (10) | .50 |
|  | Rest of Europe | 7 (1.8) | 10 (2.6) | 10 (2.6) | 27 (2.3) | 9.3 (10) | .50 |
| **Alcohol use (AUDIT**^b^**)** | | | | | | | |
|  | 0-6 | 1 (0.3) | 0 (0) | 1 (0.3) | 2 (0.2) | 2.5 (6) | .87 |
|  | 7-15 | 46 (11.9) | 45 (11.5) | 47 (12) | 138 (11.8) | 2.5 (6) | .87 |
|  | 16-19 | 72 (18.7) | 86 (22) | 76 (19.4) | 234 (20) | 2.5 (6) | .87 |
|  | 20-40 | 267 (69.2) | 260 (66.5) | 268 (68.4) | 795 (68) | 2.5 (6) | .87 |
| **Alcohol dependence (ICD-10**^c^**)** | | | | | | | |
|  | No dependence (0-2) | 41 (10.6) | 40 (10.2) | 44 (11.2) | 125 (10.7) | 0.01 (2) | .99 |
|  | Dependence (3-6) | 345 (89.4) | 351 (89.8) | 348 (88.8) | 1044 (89.3) | 0.01 (2) | .99 |
| **Alcohol use disorder (DSM-5**^d^**)** | | | | | | | |
|  | None (0-1) | 2 (0.5) | 1 (0.3) | 2 (0.5) | 5 (0.4) | 7.8 (6) | .26 |
|  | Mild (1-3) | 26 (6.7) | 11 (2.8) | 23 (5.9) | 60 (5.1) | 7.8 (6) | .26 |
|  | Moderate (4-5) | 71 (18.4) | 83 (21.2) | 75 (19.1) | 229 (19.6) | 7.8 (6) | .26 |
|  | Severe (6-12) | 287 (74.4) | 296 (75.7) | 292 (74.5) | 875 (74.9) | 7.8 (6) | .26 |
| **Anxiety (GAD-7**^e^**)** | | | | | | | |
|  | None (0-4) | 101 (26.2) | 104 (26.6) | 100 (25.5) | 305 (26.1) | 12.9 (6) | .05 |
|  | Mild (5-9) | 121 (31.3) | 146 (37.3) | 165 (42.1) | 432 (37) | 12.9 (6) | .05 |
|  | Moderate (10-14) | 98 (25.4) | 79 (20.2) | 80 (20.4) | 257 (22) | 12.9 (6) | .05 |
|  | Severe (15-21) | 66 (17.1) | 62 (15.9) | 47 (12) | 175 (15) | 12.9 (6) | .05 |
| **Depression (MADRS-S**^f^**)** | | | | | | | |
|  | None (0-12) | 99 (25.6) | 126 (32.2) | 118 (30.1) | 343 (29.3) | 10.3 (6) | .11 |
|  | Mild (13-19) | 106 (27.5) | 98 (25.1) | 115 (29.3) | 319 (27.3) | 10.3 (6) | .11 |
|  | Moderate (20-34) | 160 (41.5) | 157 (40.2) | 140 (35.7) | 457 (39.1) | 10.3 (6) | .11 |
|  | Severe (35-54) | 21 (5.4) | 10 (2.6) | 19 (4.8) | 50 (4.3) | 10.3 (6) | .11 |
| Increased risk of suicide (MADRS-S item 9 >2) | | 69 (17.9) | 70 (17.9) | 74 (18.9) | 213 (18.2) | 0.2 (2) | .89 |

^a^ICBT: internet-based cognitive behavioral therapy.

^b^AUDIT: alcohol use disorder identification test.

^c^ICD-10: International Classification of Diseases-Tenth Revision.

^d^DSM-5: Diagnostic and Statistical Manual of Mental Disorders, fifth edition.

^e^GAD-7: Generalized Anxiety Disorder Assessment-7 items.

^f^MADRS-S: Montgomery Asberg Depression Rating Scale–Self-rated.
