# Supplementary material for: Effects of Internet-Based Cognitive Behavioral Therapy for Harmful Alcohol Use and Alcohol Dependence as Self-help or With Therapist Guidance: Three-Armed Randomized Trial
Source: J Med Internet Res. 2021 Nov 24;23(11):e29666. doi: 10.2196/29666 (PMC8663526; doi:10.2196/29666)
Supplement: Multimedia Appendix 3 [file jmir_v23i11e29666_app3.docx]

Observed mean, SD, and difference between groups (N=1169).^a^

| Characteristics | | Therapist-guided  ICBT^b^ (n=386) | | Self-help  ICBT (n=391) | | Control (n=392) | | Therapist-guided  ICBT versus control | | Self-help ICBT  versus control | | Therapist-guided  ICBT versus self-help | |
| --- | --- | --- | --- | --- | --- | --- | --- | --- | --- | --- | --- | --- | --- |
|  | | Mean (SD) | n (%) | Mean (SD) | n (%) | Mean (SD) | n (%) | *t* test (*df*) | *P* value | *t* test (*df*) | *P* value | *t* test (*df*) | *P* value |
|  | |  |  |  |  |  |  |  |  |  |  |  |  |
| **Drinks per week** | | | | | | | | | | | | | |
|  | Baseline | 24.63 (16.46) | 386 (100) | 26.13 (17.52) | 391 (100) | 25.80 (17.06) | 392 (100) | 0.97 (776) | .33 | −0.26 (781) | .79 | −1.23 (775) | .22 |
|  | 3 months | 10.51 (11.74) | 200 (51.8) | 11.94 (15.93) | 183 (46.8) | 14.35 (15.73) | 219 (55.9) | 2.81 (417) | .005 | 1.52 (400) | .13 | −0.99 (381) | .32 |
|  | 6 months | 12.28 (15.27) | 169 (43.6) | 12.43 (17.04) | 153 (39.1) | 12.88 (14.44) | 186 (47.4) | 0.38 (353) | .71 | 0.27 (400) | .79 | −0.08 (321) | .94 |
| **AUDIT**^c^ | | | | | | | | | | | | | |
|  | Baseline | 22.09 (5.64) | 386 (100) | 22.23 (5.54) | 391 (100) | 22.03 (5.55) | 392 (100) | −0.17 (776) | .87 | −0.51 (781) | .61 | −0.34(775) | .74 |
|  | 3 months | 11.96 (7.16) | 200 (51.8) | 12.92 (7.60) | 183 (46.8) | 14.87 (7.56) | 219 (55.9) | 4.05 (417) | <.001 | 2.57 (400) | .01 | −1.27 (381) | .20 |
|  | 6 months | 12.28 (7.70) | 169 (43.6) | 12.86 (8.09) | 153 (39.1) | 14.29 (8.00) | 186 (47.4) | 2.41 (353) | .02 | 1.63 (338) | .10 | −0.66 (321) | .51 |
| **AUDIT-C**^d^ | | | | | | | | | | | | | |
|  | Baseline | 8.32 (1.71) | 386 (100) | 8.30 (1.79) | 391 (100) | 8.29 (1.85) | 392 (100) | −0.22 (776) | .83 | −0.05 (781) | .96 | 0.18 (775) | .86 |
|  | 3 months | 4.79 (2.96) | 200 (51.8) | 5.19 (3.03) | 183 (46.8) | 5.56 (2.94) | 219 (55.9) | 2.67 (417) | .008 | 1.24 (400) | .22 | −1.31 (381) | .19 |
|  | 6 months | 5.18 (2.98) | 169 (43.6) | 5.18 (3.10) | 153 (39.1) | 5.52 (2.98) | 186 (47.4) | 1.07 (353) | .29 | 1.01 (338) | .31 | −0.01 (321) | .99 |
| **DSM-5**^e^ | | | | | | | | | | | | | |
|  | Baseline | 7.16 (2.33) | 386 (100) | 7.25 (2.23) | 391 (100) | 7.14 (2.31) | 392 (100) | −0.17 (776) | .87 | −0.71 (781) | .48 | −0.53 (775) | .59 |
|  | 3 months | 4.61 (3.14) | 200 (51.8) | 5.03 (3.04) | 183 (46.8) | 5.37 (2.91) | 219 (55.9) | 2.54 (417) | .01 | 1.14 (400) | .25 | −1.31 (381) | .19 |
|  | 6 months | 4.70 (3.15) | 169 (43.6) | 4.89 (3.14) | 153 (39.1) | 5.35 (2.93) | 186 (47.4) | 2.01 (353) | .05 | 1.36 (338) | .17 | −0.56 (321) | .58 |
| **ICD-10**^f^ | | | | | | | | | | | | | |
|  | Baseline | 4.24 (1.33) | 386 (100) | 4.29 (1.33) | 391 (100) | 4.21 (1.37) | 392 (100) | −0.30 (776) | .76 | −0.91 (781) | .36 | −0.61 (775) | .54 |
|  | 3 months | 2.80 (1.80) | 200 (51.8) | 3.01 (1.81) | 183 (46.8) | 3.28 (1.72) | 219 (55.9) | 2.76 (417) | .006 | 1.52 (400) | .13 | −1.12 (381) | .26 |
|  | 6 months | 2.82 (1.81) | 169 (43.6) | 2.96 (1.88) | 153 (39.1) | 3.25 (1.76) | 186 (47.4) | 2.25 (353) | .02 | 1.46 (338) | .15 | −0.67 (321) | .50 |
| **MADRS-S**^g^ | | | | | | | | | | | | | |
|  | Baseline | 18.98 (9.25) | 386 (100) | 17.93 (8.87) | 391 (100) | 18.08 (9.09) | 392 (100) | −1.38 (776) | .17 | 0.22 (781) | .82 | 1.62 (775) | .11 |
|  | 3 months | 11.54 (9.32) | 200 (51.8) | 11.52 (9.14) | 183 (46.8) | 11.83 (8.83) | 219 (55.9) | 0.32 (417) | .75 | 0.34 (400) | .73 | 0.02 (381) | .98 |
|  | 6 months | 11.54 (9.63) | 169 (43.6) | 11.74 (9.75) | 153 (39.1) | 11.55 (9.18) | 186 (47.4) | 0.02 (353) | .99 | −0.18 (338) | .86 | −0.19 (321) | .85 |
| **GAD-7**^h^ | | | | | | | | | | | | | |
|  | Baseline | 8.74 (5.47) | 386 (100) | 8.19 (5.29) | 391 (100) | 7.98 (4.94) | 392 (100) | −2.05 (776) | .04 | −0.57 (781) | .57 | 1.44 (775) | .15 |
|  | 3 months | 5.29 (4.83) | 200 (51.8) | 5.36 (5.13) | 183 (46.8) | 5.56 (4.72) | 219 (55.9) | 0.57 (417) | .57 | 0.40 (400) | .69 | −0.14 (381) | .89 |
|  | 6 months | 4.89 (4.73) | 169 (43.6) | 5.69 (5.47) | 153 (39.1) | 5.35 (5.14) | 186 (47.4) | 0.87 (353) | .39 | −0.59 (338) | .56 | −1.40 (321) | .16 |
| **EQ-5D**^i^ | | | | | | | | | | | | | |
|  | Baseline | 0.71 (0.21) | 386 (100) | 0.72 (0.19) | 391 (100) | 0.74 (0.17) | 392 (100) | 2.46 (776) | .01 | 1.24 (781) | .21 | −1.23 (775) | .22 |
|  | 3 months | 0.81 (0.17) | 200 (51.8) | 0.80 (0.18) | 183 (46.8) | 0.81 (0.17) | 219 (55.9) | 0.30 (417) | .77 | 0.60 (400) | .55 | 0.31 (381) | .76 |
|  | 6 months | 0.81 (0.18) | 169 (43.6) | 0.79 (0.19) | 153 (39.1) | 0.82 (0.18) | 186 (47.4) | 0.77 (353) | .44 | 1.71 (338) | .09 | 0.98 (321) | .33 |
| **Binge drinking days** | | | | | | | | | | | | | |
|  | Baseline | 2.82 (1.88) | 386 (100) | 3.01 (2.11) | 391 (100) | 2.99 (2.08) | 392 (100) | 1.17 (776) | .24 | −0.12 (781) | .90 | −1.28 (775) | .20 |
|  | 3 months | 1.24 (1.73) | 200 (51.8) | 1.33 (1.96) | 183 (46.8) | 1.54 (1.96) | 219 (55.9) | 1.65 (417) | .10 | 1.05 (400) | .30 | −0.49 (381) | .62 |
|  | 6 months | 1.33 (1.77) | 169 (43.6) | 1.35 (1.93) | 153 (39.1) | 1.43 (1.85) | 186 (47.4) | 0.54 (353) | .59 | 0.39 (338) | .70 | −0.12 (321) | .90 |
| **Nondrinking days** | | | | | | | | | | | | | |
|  | Baseline | 2.83 (2.01) | 386 (100) | 2.74 (2.13) | 391 (100) | 2.62 (2.05) | 392 (100) | −1.45 (776) | .15 | −0.78 (781) | .44 | 0.64 (775) | .52 |
|  | 3 months | 4.53 (2.17) | 200 (51.8) | 4.28 (2.33) | 183 (46.8) | 3.99 (2.42) | 219 (55.9) | −2.40 (417) | .08 | −1.23 (400) | .22 | 1.07 (381) | .29 |
|  | 6 months | 4.37 (2.23) | 169 (43.6) | 4.33 (2.33) | 153 (39.1) | 4.15 (2.39) | 186 (47.4) | −0.91 (353) | .37 | −0.70 (338) | .48 | 0.16 (321) | .87 |
| **Drinks on drinking days** | | | | | | | | | | | | | |
|  | Baseline | 5.15 (3.84) | 386 (100) | 4.86 (3.98) | 391 (100) | 4.53 (3.63) | 392 (100) | −2.30 (776) | .02 | −1.22 (781) | .22 | 1.01 (775) | .31 |
|  | 3 months | 2.88 (2.95) | 200 (51.8) | 2.46 (2.48) | 183 (46.8) | 2.89 (3.00) | 219 (55.9) | 0.02 (417) | .99 | 1.56 (400) | .12 | 1.52 (381) | .13 |
|  | 6 months | 3.00 (3.04) | 169 (43.6) | 2.75 (2.69) | 153 (39.1) | 2.64 (2.89) | 186 (47.4) | −1.12 (353) | .26 | −0.35 (338) | .73 | 0.78 (321) | .44 |

^a^*P* values are Bonferroni-adjusted by considering *P*<.02 significant.

^b^ICBT: internet-based cognitive behavioral therapy.

^c^AUDIT: alcohol use disorder identification test.

^d^AUDIT-C: alcohol use disorder identification test consumption questions.

^e^DSM-5: Diagnostic and Statistical Manual of Mental Disorders, fifth edition.

^f^ICD-10: International Classification of Diseases, 10th Revision.

^g^MADRS-S: Montgomery Asberg Depression Rating Scale–Self-rated.

^h^GAD-7: Generalized Anxiety Disorder Assessment–7 items.

^i^EQ-5D: EuroQol-5 dimensions.
