# Supplementary material for: Effects of Internet-Based Cognitive Behavioral Therapy for Harmful Alcohol Use and Alcohol Dependence as Self-help or With Therapist Guidance: Three-Armed Randomized Trial
Source: J Med Internet Res. 2021 Nov 24;23(11):e29666. doi: 10.2196/29666 (PMC8663526; doi:10.2196/29666)
Supplement: Multimedia Appendix 4 [file jmir_v23i11e29666_app4.docx]

| **Outcome** | **Follow-up** | **Contrast** | **Imputed range** | **Tipping point mean in control group assuming MAR in treatment group** | **Tipping point mean in treatment group assuming MAR in control group** |
| --- | --- | --- | --- | --- | --- |
| AUDIT | 3-month | Control vs self-help | 0-36 | 13.3 | 14.2 |
| AUDIT | 3-month | Control vs guided | 0-36 | 11.2 | 15.4 |
| AUDIT-C | 3-month | Control vs guided | 0-12 | 4.9 | 5.4 |
| DSM criteria | 3-month | Control vs guided | 0-11 | 4.8 | 5.2 |
| ICD criteria | 3-month | Control vs guided | 0-6 | 2.8 | 3.2 |
