# Supplementary material for: Effects of Internet-Based Cognitive Behavioral Therapy for Harmful Alcohol Use and Alcohol Dependence as Self-help or With Therapist Guidance: Three-Armed Randomized Trial
Source: J Med Internet Res. 2021 Nov 24;23(11):e29666. doi: 10.2196/29666 (PMC8663526; doi:10.2196/29666)
Supplement: Multimedia Appendix 5 [file jmir_v23i11e29666_app5.docx]

|  | MADRS^§^ | | | GAD-7^§^ | | | EQ5D^§^ | | | Sober days^§^ | | | Drinks a drinking day* | | | Binge drinking days^†^ | | |
| --- | --- | --- | --- | --- | --- | --- | --- | --- | --- | --- | --- | --- | --- | --- | --- | --- | --- | --- |
|  | B | S E | *P* | B | S E | *P* | B | S E | *P* | B | S E | *P* | B | S E | *P* | B | S E | *P* |
| (intercept) | 18.08 | 0.45 | 0.000 | 7.98 | 0.26 | 0.000 | 0.74 | 0.01 | 0.000 | 2.62 | 0.11 | 0.000 | 0.50 | 0.11 | 0.000 | 0.99 | 0.04 | 0.000 |
| time | -8.74 | 1.00 | 0.000 | -3.20 | 0.59 | 0.000 | 0.09 | 0.02 | 0.000 | 1.99 | 0.26 | 0.000 | -0.65 | 0.29 | 0.023 | -1.13 | 0.13 | 0.000 |
| time^2^ | 2.84 | 0.50 | 0.000 | 1.01 | 0.29 | 0.001 | -0.03 | 0.01 | 0.005 | -0.63 | 0.13 | 0.000 | 0.06 | 0.15 | 0.690 | 0.27 | 0.07 | 0.000 |
| therapist | 0.91 | 0.64 | 0.157 | 0.77 | 0.37 | 0.038 | -0.03 | 0.01 | 0.012 | 0.21 | 0.15 | 0.160 | 0.33 | 0.16 | 0.038 | -0.04 | 0.06 | 0.446 |
| self-help | -0.14 | 0.64 | 0.823 | 0.21 | 0.37 | 0.568 | -0.02 | 0.01 | 0.232 | 0.12 | 0.15 | 0.437 | 0.10 | 0.16 | 0.538 | 0.00 | 0.06 | 0.975 |
| therapist x time | -1.90 | 1.44 | 0.188 | -1.08 | 0.85 | 0.203 | 0.04 | 0.03 | 0.100 | 0.59 | 0.37 | 0.112 | -0.51 | 0.40 | 0.210 | -0.29 | 0.20 | 0.137 |
| therapist x time^2^ | 0.70 | 0.72 | 0.334 | 0.27 | 0.43 | 0.524 | -0.02 | 0.01 | 0.204 | -0.29 | 0.19 | 0.122 | 0.27 | 0.21 | 0.191 | 0.15 | 0.10 | 0.133 |
| self-help x time | -0.06 | 1.48 | 0.968 | -0.23 | 0.87 | 0.791 | 0.01 | 0.03 | 0.734 | 0.24 | 0.38 | 0.523 | -0.62 | 0.41 | 0.135 | -0.23 | 0.20 | 0.263 |
| self-help x time^2^ | 0.15 | 0.75 | 0.837 | 0.14 | 0.44 | 0.749 | -0.01 | 0.01 | 0.619 | -0.13 | 0.19 | 0.512 | 0.36 | 0.21 | 0.084 | 0.10 | 0.11 | 0.365 |

Time was coded in 3-month-periods (0. 1 and 2). Reference group was Control. ^*^ Generalized linear mixed model. Neg-binominal distribution. dispersion coefficient: 2.407. ^†^ Generalized linear mixed model. Neg-binominal distribution. dispersion coefficient: 0.415. Covariance structure for random effects Variance component and for repeated effects Diagonal. ^§^ Linear mixed model. Covariance structure for random effects Variance component and for repeated effects First-Order Autoregressive.
