# Supplementary material for: Effects of Internet-Based Cognitive Behavioral Therapy for Harmful Alcohol Use and Alcohol Dependence as Self-help or With Therapist Guidance: Three-Armed Randomized Trial
Source: J Med Internet Res. 2021 Nov 24;23(11):e29666. doi: 10.2196/29666 (PMC8663526; doi:10.2196/29666)
Supplement: Multimedia Appendix 6 [file jmir_v23i11e29666_app6.docx]

**Correlations between changes in outcomes at follow-ups**


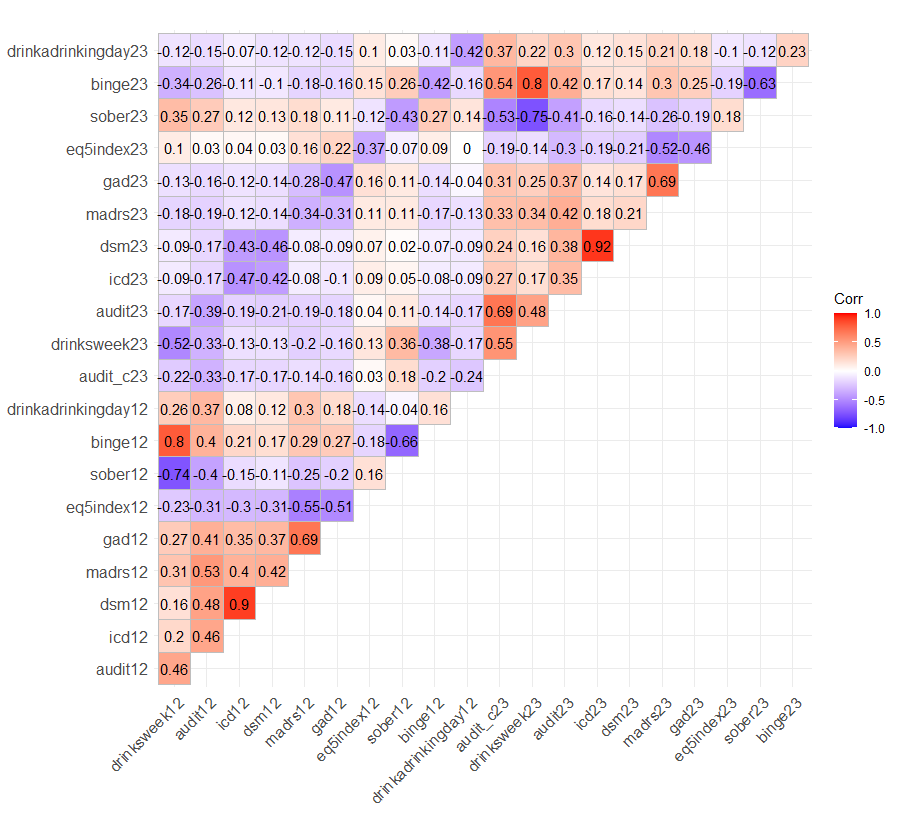


[outcome variable]12 : Change in outcome between baseline and the first follow-up at 3 months

[outcome variable]23 : Change in outcome between the first follow-up at 3 months and the second follow-up at 6 months
