# Supplementary material for: Effects of Internet-Based Cognitive Behavioral Therapy for Harmful Alcohol Use and Alcohol Dependence as Self-help or With Therapist Guidance: Three-Armed Randomized Trial
Source: J Med Internet Res. 2021 Nov 24;23(11):e29666. doi: 10.2196/29666 (PMC8663526; doi:10.2196/29666)
Supplement: Multimedia Appendix 7 [file jmir_v23i11e29666_app7.pdf]

CONSORT-EHEALTH (V 1.6.1) - Submission/Publication Form

The CONSORT-EHEALTH checklist is intended for authors of randomized trials evaluating web-based and Internet-based applications/interventions, including mobile interventions, electronic games (incl multiplayer games), social media, certain telehealth applications, and other interactive and/or networked electronic applications. Some of the items (e.g. all subitems under item 5 - description of the intervention) may also be applicable for other study designs.

The goal of the CONSORT EHEALTH checklist and guideline is to be
a) a guide for reporting for authors of RCTs,
b) to form a basis for appraisal of an ehealth trial (in terms of validity)

CONSORT-EHEALTH items/subitems are MANDATORY reporting items for studies published in the Journal of Medical Internet Research and other journals / scientific societies endorsing the checklist.

Items numbered 1., 2., 3., 4a., 4b etc are original CONSORT or CONSORT-NPT (non-pharmacologic treatment) items.
Items with Roman numerals (i., ii, iii, iv etc.) are CONSORT-EHEALTH extensions/clarifications.

As the CONSORT-EHEALTH checklist is still considered in a formative stage, we would ask that you also RATE ON A SCALE OF 1-5 how important/useful you feel each item is FOR THE PURPOSE OF THE CHECKLIST and reporting guideline (optional).

Mandatory reporting items are marked with a red *.
In the textboxes, either copy & paste the relevant sections from your manuscript into this form - please include any quotes from your manuscript in QUOTATION MARKS,
or answer directly by providing additional information not in the manuscript, or elaborating on why the item was not relevant for this study.

YOUR ANSWERS WILL BE PUBLISHED AS A SUPPLEMENTARY FILE TO YOUR PUBLICATION IN JMIR AND ARE CONSIDERED PART OF YOUR PUBLICATION (IF ACCEPTED).
Please fill in these questions diligently. Information will not be copyedited, so please use proper spelling and grammar, use correct capitalization, and avoid abbreviations.

DO NOT FORGET TO SAVE AS PDF _AND_ CLICK THE SUBMIT BUTTON SO YOUR ANSWERS ARE IN OUR DATABASE !!!

Ditt svar är för stort. Testa att förkorta en del av dina svar.

Eysenbach G, CONSORT-EHEALTH Group

CONSORT-EHEALTH: Improving and Standardizing Evaluation Reports of Web-based and Mobile Health Interventions
J Med Internet Res 2011;13(4):e126
URL: http://www.jmir.org/2011/4/e126/
doi: 10.2196/jmir.1923
PMID: 22209829

*Obligatorisk

Your name *

First Last

Magnus Johansson

Primary Affiliation (short), City, Country *

University of Toronto, Toronto, Canada

Karolinska Intstitutet

Your e-mail address *

abc@gmail.com

magnus.johansson.1@ki.se

Title of your manuscript *

Provide the (draft) title of your manuscript.

Web-based treatment of harmful alcohol use and alcohol dependence: A randomized controlled trial

Ditt svar är för stort. Testa att förkorta en del av dina svar.

Name of your App/Software/Intervention *

If there is a short and a long/alternate name, write the short name first and add the long name in brackets.

Alkoholhjälpen

Evaluated Version (if any)

e.g. "V1", "Release 2017-03-01", "Version 2.0.27913"

V4, Release 2015-03-01

Language(s) *

What language is the intervention/app in? If multiple languages are available, separate by comma (e.g. "English, French")

Swedish

URL of your Intervention Website or App

e.g. a direct link to the mobile app on app in appstore (itunes, Google Play), or URL of the website. If the intervention is a DVD or hardware, you can also link to an Amazon page.

https://alkoholhjalpen.se/

URL of an image/screenshot (optional)

Ditt svar

Ditt svar är för stort. Testa att förkorta en del av dina svar.

Accessibility *

Can an enduser access the intervention presently?

- (•) access is free and open
- ( ) access only for special usergroups, not open
- ( ) access is open to everyone, but requires payment/subscription/in-app purchases
- ( ) app/intervention no longer accessible
- ( ) Övrigt:

Primary Medical Indication/Disease/Condition *

e.g. "Stress", "Diabetes", or define the target group in brackets after the condition, e.g. "Autism (Parents of children with)", "Alzheimers (Informal Caregivers of)"

Alcohol use disorder

Primary Outcomes measured in trial *

comma-separated list of primary outcomes reported in the trial

weekly standard drinks

Secondary/other outcomes

Are there any other outcomes the intervention is expected to affect?

AUDIT, diagnostic criteria, GAD-7, MADRS-S

Ditt svar är för stort. Testa att förkorta en del av dina svar.

Recommended "Dose" *

What do the instructions for users say on how often the app should be used?

- ○ Approximately Daily
- ◉ Approximately Weekly
- ○ Approximately Monthly
- ○ Approximately Yearly
- ○ "as needed"
- ○ Övrigt:

Approx. Percentage of Users (starters) still using the app as recommended after 3 months *

- ○ unknown / not evaluated
- ○ 0-10%
- ○ 11-20%
- ○ 21-30%
- ◉ 31-40%
- ○ 41-50%
- ○ 51-60%
- ○ 61-70%
- ○ 71%-80%
- ○ 81-90%
- ○ 91-100%

Ditt svar är för stort. Testa att förkorta en del av dina svar.

Overall, was the app/intervention effective? *

- ( ) yes: all primary outcomes were significantly better in intervention group vs control
- (•) partly: SOME primary outcomes were significantly better in intervention group vs control
- ( ) no statistically significant difference between control and intervention
- ( ) potentially harmful: control was significantly better than intervention in one or more outcomes
- ( ) inconclusive: more research is needed
- ( ) Övrigt:

Article Preparation Status/Stage *

At which stage in your article preparation are you currently (at the time you fill in this form)

- ( ) not submitted yet - in early draft status
- ( ) not submitted yet - in late draft status, just before submission
- (•) submitted to a journal but not reviewed yet
- ( ) submitted to a journal and after receiving initial reviewer comments
- ( ) submitted to a journal and accepted, but not published yet
- ( ) published
- ( ) Övrigt:

Ditt svar är för stort. Testa att förkorta en del av dina svar.

Journal *

If you already know where you will submit this paper (or if it is already submitted), please provide the journal name (if it is not JMIR, provide the journal name under "other")

- ○ not submitted yet / unclear where I will submit this
- ◉ Journal of Medical Internet Research (JMIR)
- ○ JMIR mHealth and UHealth
- ○ JMIR Serious Games
- ○ JMIR Mental Health
- ○ JMIR Public Health
- ○ JMIR Formative Research
- ○ Other JMIR sister journal
- ○ Övrigt:

Is this a full powered effectiveness trial or a pilot/feasibility trial? *

- ○ Pilot/feasibility
- ◉ Fully powered

Manuscript tracking number *

If this is a JMIR submission, please provide the manuscript tracking number under "other" (The ms tracking number can be found in the submission acknowledgement email, or when you login as author in JMIR. If the paper is already published in JMIR, then the ms tracking number is the four-digit number at the end of the DOI, to be found at the bottom of each published article in JMIR)

- ○ no ms number (yet) / not (yet) submitted to / published in JMIR
- ◉ Övrigt: 29666

Ditt svar är för stort. Testa att förkorta en del av dina svar.

TITLE AND ABSTRACT

1a) TITLE: Identification as a randomized trial in the title

1a) Does your paper address CONSORT item 1a? *

I.e does the title contain the phrase "Randomized Controlled Trial"? (if not, explain the reason under "other")

- (x) yes
- ( ) Övrigt:

1a-i) Identify the mode of delivery in the title

Identify the mode of delivery. Preferably use “web-based” and/or “mobile” and/or “electronic game” in the title. Avoid ambiguous terms like “online”, “virtual”, “interactive”. Use “Internet-based” only if Intervention includes non-web-based Internet components (e.g. email), use “computer-based” or “electronic” only if offline products are used. Use “virtual” only in the context of “virtual reality” (3-D worlds). Use “online” only in the context of “online support groups”. Complement or substitute product names with broader terms for the class of products (such as “mobile” or “smart phone” instead of “iphone”), especially if the application runs on different platforms.

	1	2	3	4	5	
subitem not at all important	( )	( )	( )	( )	(x)	essential

Rensa markering

Does your paper address subitem 1a-i? *

Copy and paste relevant sections from manuscript title (include quotes in quotation marks "like this" to indicate direct quotes from your manuscript), or elaborate on this item by providing additional information not in the ms, or briefly explain why the item is not applicable/relevant for your study

"Web-based treatment of harmful alcohol use and alcohol dependence: A randomized

Ditt svar är för stort. Testa att förkorta en del av dina svar.

1a-ii) Non-web-based components or important co-interventions in title

Mention non-web-based components or important co-interventions in title, if any (e.g., “with telephone support”).

	1	2	3	4	5	
subitem not at all important	○	○	○	◉	○	essential

Rensa markering

Does your paper address subitem 1a-ii?

Copy and paste relevant sections from manuscript title (include quotes in quotation marks "like this" to indicate direct quotes from your manuscript), or elaborate on this item by providing additional information not in the ms, or briefly explain why the item is not applicable/relevant for your study

Not applicable: no non-web-based components were used in the interventions

1a-iii) Primary condition or target group in the title

Mention primary condition or target group in the title, if any (e.g., “for children with Type I Diabetes”) Example: A Web-based and Mobile Intervention with Telephone Support for Children with Type I Diabetes: Randomized Controlled Trial

	1	2	3	4	5	
subitem not at all important	○	○	○	○	◉	essential

Rensa markering

Does your paper address subitem 1a-iii? *

Copy and paste relevant sections from manuscript title (include quotes in quotation marks "like this" to indicate direct quotes from your manuscript), or elaborate on this item by providing additional information not in the ms, or briefly explain why the item is not applicable/relevant for your study

[illegible]

Ditt svar är för stort. Testa att förkorta en del av dina svar.

1b) ABSTRACT: Structured summary of trial design, methods, results, and conclusions

NPT extension: Description of experimental treatment, comparator, care providers, centers, and blinding status.

1b-i) Key features/functionalities/components of the intervention and comparator in the METHODS section of the ABSTRACT

Mention key features/functionalities/components of the intervention and comparator in the abstract. If possible, also mention theories and principles used for designing the site. Keep in mind the needs of systematic reviewers and indexers by including important synonyms. (Note: Only report in the abstract what the main paper is reporting. If this information is missing from the main body of text, consider adding it)

	1	2	3	4	5	
subitem not at all important	○	○	○	◉	○	essential

Rensa markering

Does your paper address subitem 1b-i? *

Copy and paste relevant sections from the manuscript abstract (include quotes in quotation marks "like this" to indicate direct quotes from your manuscript), or elaborate on this item by providing additional information not in the ms, or briefly explain why the item is not applicable/relevant for your study

"comparing therapist-guided ICBT and self-help ICBT with an information only control condition"

"Participants in the therapist-guided ICBT and self-help ICBT groups had 12-week access to a program consisting of five main and three extra modules as well as a drinking-calendar with automatic feedback."

Ditt svar är för stort. Testa att förkorta en del av dina svar.

1b-ii) Level of human involvement in the METHODS section of the ABSTRACT

Clarify the level of human involvement in the abstract, e.g., use phrases like “fully automated” vs. “therapist/nurse/care provider/physician-assisted” (mention number and expertise of providers involved, if any). (Note: Only report in the abstract what the main paper is reporting. If this information is missing from the main body of text, consider adding it)

	1	2	3	4	5	
subitem not at all important	○	○	○	●	○	essential

Rensa markering

Does your paper address subitem 1b-ii?

Copy and paste relevant sections from the manuscript abstract (include quotes in quotation marks "like this" to indicate direct quotes from your manuscript), or elaborate on this item by providing additional information not in the ms, or briefly explain why the item is not applicable/relevant for your study

"therapist-guided ICBT"

"The guidance was given by experienced therapists trained in motivational interviewing"

1b-iii) Open vs. closed, web-based (self-assessment) vs. face-to-face assessments in the METHODS section of the ABSTRACT

Mention how participants were recruited (online vs. offline), e.g., from an open access website or from a clinic or a closed online user group (closed usergroup trial), and clarify if this was a purely web-based trial, or there were face-to-face components (as part of the intervention or for assessment). Clearly say if outcomes were self-assessed through questionnaires (as common in web-based trials). Note: In traditional offline trials, an open trial (open-label trial) is a type of clinical trial in which both the researchers and participants know which treatment is being administered. To avoid confusion, use “blinded” or “unblinded” to indicated the level of blinding instead of “open”, as “open” in web-based trials usually refers to “open access” (i.e. participants can self-enrol). (Note: Only report in the abstract what the main paper is reporting. If this information is missing from the main body of text, consider adding it)

	1	2	3	4	5	
subitem not at all important	○	○	●	○	○	essential

Rensa markering

Ditt svar är för stort. Testa att förkorta en del av dina svar.

Does your paper address subitem 1b-iii?

Copy and paste relevant sections from the manuscript abstract (include quotes in quotation marks "like this" to indicate direct quotes from your manuscript), or elaborate on this item by providing additional information not in the ms, or briefly explain why the item is not applicable/relevant for your study

"Help-seeking adult internet-users" details on online-recruitment, self-assessed outcomes and blinding is included in manuscript, but not in abstract

1b-iv) RESULTS section in abstract must contain use data

Report number of participants enrolled/assessed in each group, the use/uptake of the intervention (e.g., attrition/adherence metrics, use over time, number of logins etc.), in addition to primary/secondary outcomes. (Note: Only report in the abstract what the main paper is reporting. If this information is missing from the main body of text, consider adding it)

	1	2	3	4	5	
subitem not at all important	○	○	◉	○	○	essential

Rensa markering

Does your paper address subitem 1b-iv?

Copy and paste relevant sections from the manuscript abstract (include quotes in quotation marks "like this" to indicate direct quotes from your manuscript), or elaborate on this item by providing additional information not in the ms, or briefly explain why the item is not applicable/relevant for your study

"internet-users (n=1169)" Attrition/adherence metrics only in main manuscript.

Ditt svar är för stort. Testa att förkorta en del av dina svar.

1b-v) CONCLUSIONS/DISCUSSION in abstract for negative trials

Conclusions/Discussions in abstract for negative trials: Discuss the primary outcome - if the trial is negative (primary outcome not changed), and the intervention was not used, discuss whether negative results are attributable to lack of uptake and discuss reasons. (Note: Only report in the abstract what the main paper is reporting. If this information is missing from the main body of text, consider adding it)

	1	2	3	4	5	
subitem not at all important	○	○	○	◉	○	essential

Rensa markering

Does your paper address subitem 1b-v?

Copy and paste relevant sections from the manuscript abstract (include quotes in quotation marks "like this" to indicate direct quotes from your manuscript), or elaborate on this item by providing additional information not in the ms, or briefly explain why the item is not applicable/relevant for your study

"we did not find a therapist-guided ICBT program to be more effective than the same program as self-help ICBT in reducing alcohol consumption or other alcohol-related outcomes. In the short run therapist-guided ICBT seems be more effective than information."

INTRODUCTION

2a) In INTRODUCTION: Scientific background and explanation of rationale

Ditt svar är för stort. Testa att förkorta en del av dina svar.

2a-i) Problem and the type of system/solution

Describe the problem and the type of system/solution that is object of the study: intended as stand-alone intervention vs. incorporated in broader health care program? Intended for a particular patient population? Goals of the intervention, e.g., being more cost-effective to other interventions, replace or complement other solutions? (Note: Details about the intervention are provided in “Methods” under 5)

	1	2	3	4	5	
subitem not at all important	○	○	○	◉	○	essential

Rensa markering

Does your paper address subitem 2a-i? *

Copy and paste relevant sections from the manuscript (include quotes in quotation marks "like this" to indicate direct quotes from your manuscript), or elaborate on this item by providing additional information not in the ms, or briefly explain why the item is not applicable/relevant for your study

"Internet interventions can help overcome some of the problems with implementation, limited accessibility and stigma"

"More extended internet alcohol interventions are intended to be used over a number of weeks or sessions"

"Therapist guided ICBT for alcohol were tested in five of the 14 previous studies and was found to be more effective than waiting-list"

"need for studies on internet interventions aimed specifically at people with harmful use or alcohol dependence [19], since many of the participants in previous studies of ICBT programs for alcohol have had AUDIT-scores indicating a higher level of alcohol related problems."

Ditt svar är för stort. Testa att förkorta en del av dina svar.

2a-ii) Scientific background, rationale: What is known about the (type of) system

Scientific background, rationale: What is known about the (type of) system that is the object of the study (be sure to discuss the use of similar systems for other conditions/diagnoses, if appropiate), motivation for the study, i.e. what are the reasons for and what is the context for this specific study, from which stakeholder viewpoint is the study performed, potential impact of findings [2]. Briefly justify the choice of the comparator.

	1	2	3	4	5	
subitem not at all important	○	○	○	○	○	essential

Does your paper address subitem 2a-ii? *

Copy and paste relevant sections from the manuscript (include quotes in quotation marks "like this" to indicate direct quotes from your manuscript), or elaborate on this item by providing additional information not in the ms, or briefly explain why the item is not applicable/relevant for your study

"the effects of ICBT programs as well as therapist guided ICBT for alcohol are still unclear. There is also a need for studies on internet interventions aimed specifically at people with harmful use or alcohol dependence"

"In a Cochrane review on digital alcohol interventions, including internet interventions (in 37 of 57 studies), the effect compared to no or minimal interventions was 23 grams (95% CI 15 to 30) less alcohol consumed weekly. According to a recent individual patient data meta-analysis of internet alcohol interventions, the effect on alcohol consumption compared to various controls were -22 grams per week (95 % CI -8.7 to -34.6) [18]."

2b) In INTRODUCTION: Specific objectives or hypotheses

Ditt svar är för stort. Testa att förkorta en del av dina svar.

Does your paper address CONSORT subitem 2b? *

Copy and paste relevant sections from the manuscript (include quotes in quotation marks "like this" to indicate direct quotes from your manuscript), or elaborate on this item by providing additional information not in the ms, or briefly explain why the item is not applicable/relevant for your study

"The hypothesis of the trial were that:
1) a therapist-guided ICBT program would lead to a greater reduction in alcohol consumption and in alcohol related problems than information alone.
2) a self-help ICBT program would lead to a greater reduction in alcohol consumption and in alcohol related problems than information alone.
3) a therapist-guided ICBT program would lead to a greater reduction in alcohol consumption and in alcohol related problems than a self-help ICBT program."

METHODS

3a) Description of trial design (such as parallel, factorial) including allocation ratio

Does your paper address CONSORT subitem 3a? *

Copy and paste relevant sections from the manuscript (include quotes in quotation marks "like this" to indicate direct quotes from your manuscript), or elaborate on this item by providing additional information not in the ms, or briefly explain why the item is not applicable/relevant for your study

"In a 3-arm randomized controlled trial, with a parallel design, participants were randomly assigned to an Internet-delivered cognitive-behavioral therapy (ICBT) program as self-help, with therapist-guidance or to information control at a ratio of 1:1:1 and block size of 30."

3b) Important changes to methods after trial commencement (such as eligibility criteria), with reasons

Ditt svar är för stort. Testa att förkorta en del av dina svar.

Does your paper address CONSORT subitem 3b? *

Copy and paste relevant sections from the manuscript (include quotes in quotation marks "like this" to indicate direct quotes from your manuscript), or elaborate on this item by providing additional information not in the ms, or briefly explain why the item is not applicable/relevant for your study

Not currently included in manuscript. No such changes were made.

3b-i) Bug fixes, Downtimes, Content Changes

Bug fixes, Downtimes, Content Changes: ehealth systems are often dynamic systems. A description of changes to methods therefore also includes important changes made on the intervention or comparator during the trial (e.g., major bug fixes or changes in the functionality or content) (5-iii) and other “unexpected events” that may have influenced study design such as staff changes, system failures/downtimes, etc. [2].

	1	2	3	4	5	
subitem not at all important	○	◉	○	○	○	essential

Rensa markering

Does your paper address subitem 3b-i?

Copy and paste relevant sections from the manuscript (include quotes in quotation marks "like this" to indicate direct quotes from your manuscript), or elaborate on this item by providing additional information not in the ms, or briefly explain why the item is not applicable/relevant for your study

Not currently included in manuscript. There were no changes in content or downtimes, during the study. Only small bugfixes and security updates to the platform were made.

4a) Eligibility criteria for participants

Ditt svar är för stort. Testa att förkorta en del av dina svar.

Does your paper address CONSORT subitem 4a? *

Copy and paste relevant sections from the manuscript (include quotes in quotation marks "like this" to indicate direct quotes from your manuscript), or elaborate on this item by providing additional information not in the ms, or briefly explain why the item is not applicable/relevant for your study

"Individuals were included if they had harmful use (defined as >15 total score on the AUDIT) or alcohol dependence (confirmed 3 or more ICD-10 criteria)."

4a-i) Computer / Internet literacy

Computer / Internet literacy is often an implicit “de facto” eligibility criterion - this should be explicitly clarified.

	1	2	3	4	5	
subitem not at all important	○	○	◉	○	○	essential

Rensa markering

Does your paper address subitem 4a-i?

Copy and paste relevant sections from the manuscript (include quotes in quotation marks "like this" to indicate direct quotes from your manuscript), or elaborate on this item by providing additional information not in the ms, or briefly explain why the item is not applicable/relevant for your study

"To be able to complete the registration the participant needed to understand written Swedish and be computer literate enough to access and navigate the website via a computer, tablet or smartphone."

Ditt svar är för stort. Testa att förkorta en del av dina svar.

4a-ii) Open vs. closed, web-based vs. face-to-face assessments:

Open vs. closed, web-based vs. face-to-face assessments: Mention how participants were recruited (online vs. offline), e.g., from an open access website or from a clinic, and clarify if this was a purely web-based trial, or there were face-to-face components (as part of the intervention or for assessment), i.e., to what degree got the study team to know the participant. In online-only trials, clarify if participants were quasi-anonymous and whether having multiple identities was possible or whether technical or logistical measures (e.g., cookies, email confirmation, phone calls) were used to detect/prevent these.

	1	2	3	4	5	
subitem not at all important	○	○	○	●	○	essential

Rensa markering

Does your paper address subitem 4a-ii? *

Copy and paste relevant sections from the manuscript (include quotes in quotation marks "like this" to indicate direct quotes from your manuscript), or elaborate on this item by providing additional information not in the ms, or briefly explain why the item is not applicable/relevant for your study

"Participants were recruited at the Swedish internet site alkoholhjälpen.se"

"Eligible participants were asked to complete online baseline questionnaires"

4a-iii) Information giving during recruitment

Information given during recruitment. Specify how participants were briefed for recruitment and in the informed consent procedures (e.g., publish the informed consent documentation as appendix, see also item X26), as this information may have an effect on user self-selection, user expectation and may also bias results.

	1	2	3	4	5	
subitem not at all important	○	○	○	●	○	essential

Rensa markering

Ditt svar är för stort. Testa att förkorta en del av dina svar.

Does your paper address subitem 4a-iii?

Copy and paste relevant sections from the manuscript (include quotes in quotation marks "like this" to indicate direct quotes from your manuscript), or elaborate on this item by providing additional information not in the ms, or briefly explain why the item is not applicable/relevant for your study

"Interested users were informed that they would answer a survey and be randomized to one of three different forms of support, but were not informed about the specifics. Adult individuals who gave their informed consent were instructed to create a personal account, with a unique username and password. They were then directed to a screening page where they were required to give informed consent for participation in the study"

"Participants were blinded to what kind of support that participants in the other groups received."

4b) Settings and locations where the data were collected

Does your paper address CONSORT subitem 4b? *

Copy and paste relevant sections from the manuscript (include quotes in quotation marks "like this" to indicate direct quotes from your manuscript), or elaborate on this item by providing additional information not in the ms, or briefly explain why the item is not applicable/relevant for your study

"Participants were recruited at the Swedish internet site alkoholhjälpen.se, an open access website that provides information and a discussion forum to individuals seeking help online for alcohol consumption. The site has been publicly accessible since 2007. During the recruitment period for the current study, alkoholhjälpen.se had approximately 20 000 unique visitors every month and a mean of 100 new forum-posts every day."

"All visitors on alkoholhjälpen.se from March 2015 to March 2017 were invited to participate in a study to develop and test different forms of internet-delivered support for changing alcohol habits"

"Eligible participants were asked to complete online baseline questionnaires including primary and secondary measures (see Measures). All participants were treated as anonymous users. They were asked to provide an email-address and a mobile phone number for notifications and follow-up reminders. Email and phone numbers were not verified, nor used for identification or for any other purposes."

Ditt svar är för stort. Testa att förkorta en del av dina svar.

4b-i) Report if outcomes were (self-)assessed through online questionnaires

Clearly report if outcomes were (self-)assessed through online questionnaires (as common in web-based trials) or otherwise.

	1	2	3	4	5	
subitem not at all important	○	○	○	◉	○	essential

Rensa markering

Does your paper address subitem 4b-i? *

Copy and paste relevant sections from the manuscript (include quotes in quotation marks "like this" to indicate direct quotes from your manuscript), or elaborate on this item by providing additional information not in the ms, or briefly explain why the item is not applicable/relevant for your study

"At follow-up participants were e-mailed a link, or re-directed when logging in on the intervention website, to the follow-up questionnaires."

4b-ii) Report how institutional affiliations are displayed

Report how institutional affiliations are displayed to potential participants [on ehealth media], as affiliations with prestigious hospitals or universities may affect volunteer rates, use, and reactions with regards to an intervention.(Not a required item – describe only if this may bias results)

	1	2	3	4	5	
subitem not at all important	○	◉	○	○	○	essential

Rensa markering

Ditt svar är för stort. Testa att förkorta en del av dina svar.

Does your paper address subitem 4b-ii?

Copy and paste relevant sections from the manuscript (include quotes in quotation marks "like this" to indicate direct quotes from your manuscript), or elaborate on this item by providing additional information not in the ms, or briefly explain why the item is not applicable/relevant for your study

Not currently included in manuscript. alkoholhjalpen.se is run by Stockholm Center of Dependency disorders with funds from the Swedish public health authorites, which was stated in the footer of the site. The study was conducted by Karoliska Institute, which was stated in the informed consent. This was the same for all interventions groups.

5) The interventions for each group with sufficient details to allow replication, including how and when they were actually administered

5-i) Mention names, credential, affiliations of the developers, sponsors, and owners

Mention names, credential, affiliations of the developers, sponsors, and owners [6] (if authors/evaluators are owners or developer of the software, this needs to be declared in a “Conflict of interest” section or mentioned elsewhere in the manuscript).

	1	2	3	4	5	
subitem not at all important	○	○	○	◉	○	essential

Rensa markering

Does your paper address subitem 5-i?

Copy and paste relevant sections from the manuscript (include quotes in quotation marks "like this" to indicate direct quotes from your manuscript), or elaborate on this item by providing additional information not in the ms, or briefly explain why the item is not applicable/relevant for your study

Not currently included in manuscript. The first author (MJ) has configured the internet platform and co-authored the program used in the study. The program is licensed under creative commons and the internet platform Drupal is open-source. MJ, PL and MG are employed at the Addiction eClinic, both part of the Stockholm Center of Dependency [illegible]

Ditt svar är för stort. Testa att förkorta en del av dina svar.

5-ii) Describe the history/development process

Describe the history/development process of the application and previous formative evaluations (e.g., focus groups, usability testing), as these will have an impact on adoption/use rates and help with interpreting results.

	1	2	3	4	5	
subitem not at all important	○	◉	○	○	○	essential

Rensa markering

Does your paper address subitem 5-ii?

Copy and paste relevant sections from the manuscript (include quotes in quotation marks "like this" to indicate direct quotes from your manuscript), or elaborate on this item by providing additional information not in the ms, or briefly explain why the item is not applicable/relevant for your study

Not currently included in manuscript. The first version of the iCBT program at alkoholhjalpen.se was developed 2007 in co-operation with an advertising and communication agency. It was programed in Flash with integrated animations and videos. The development included focus-groups of potential users and the program was one of the interventions tested in a controlled trial (Sinadinovic et al. 2014). The second version moved the program into the content management system (CMS) Drupal (drupal.org). The third version (eChange) was a shorter program, inspired by the study by Blankers et al (Blankers, Koeter, and Schippers 2011), which was tested in a pilot (Sundstrom et al. 2016) and an observational study (Johansson, Sinadinovic, Hammarberg, Sundström, et al. 2017). The Fourth and current version (eChange alkoholhjälpen) is based on previous programs and updated based on the feedback from users and experience from previous studies.

Ditt svar är för stort. Testa att förkorta en del av dina svar.

5-iii) Revisions and updating

Revisions and updating. Clearly mention the date and/or version number of the application/intervention (and comparator, if applicable) evaluated, or describe whether the intervention underwent major changes during the evaluation process, or whether the development and/or content was "frozen" during the trial. Describe dynamic components such as news feeds or changing content which may have an impact on the replicability of the intervention (for unexpected events see item 3b).

	1	2	3	4	5	
subitem not at all important	○	◉	○	○	○	essential

Rensa markering

Does your paper address subitem 5-iii?

Copy and paste relevant sections from the manuscript (include quotes in quotation marks "like this" to indicate direct quotes from your manuscript), or elaborate on this item by providing additional information not in the ms, or briefly explain why the item is not applicable/relevant for your study

Not currently included in manuscript. Development of the service was frozen during the study. The only changes were new content posted by users in the discussion forum and a news feed. Minor technical updates were also made to keep the platform secure.

5-iv) Quality assurance methods

Provide information on quality assurance methods to ensure accuracy and quality of information provided [1], if applicable.

	1	2	3	4	5	
subitem not at all important	○	○	○	◉	○	essential

Rensa markering

Ditt svar är för stort. Testa att förkorta en del av dina svar.

Does your paper address subitem 5-iv?

Copy and paste relevant sections from the manuscript (include quotes in quotation marks "like this" to indicate direct quotes from your manuscript), or elaborate on this item by providing additional information not in the ms, or briefly explain why the item is not applicable/relevant for your study

"The three therapists involved had several years' experience from the Swedish alcohol help-line [47] and had all reached approved level in phone based motivational interviewing prior to entering the study. They had been trained in ICBT and received regular supervision from the first author, who is a trained therapist with several years' experience of work with CBT and ICBT programs. "

5-v) Ensure replicability by publishing the source code, and/or providing screenshots/screen-capture video, and/or providing flowcharts of the algorithms used

Ensure replicability by publishing the source code, and/or providing screenshots/screen-capture video, and/or providing flowcharts of the algorithms used. Replicability (i.e., other researchers should in principle be able to replicate the study) is a hallmark of scientific reporting.

	1	2	3	4	5	
subitem not at all important	○	○	◉	○	○	essential

Rensa markering

Does your paper address subitem 5-v?

Copy and paste relevant sections from the manuscript (include quotes in quotation marks "like this" to indicate direct quotes from your manuscript), or elaborate on this item by providing additional information not in the ms, or briefly explain why the item is not applicable/relevant for your study

Screenshots included in manuscript. Snapshots and content of previous and current versions are available via the first author. The Swedish program content is licensed according to creative commons attribution share alike (creativecommons.org).

Ditt svar är för stort. Testa att förkorta en del av dina svar.

5-vi) Digital preservation

Digital preservation: Provide the URL of the application, but as the intervention is likely to change or disappear over the course of the years; also make sure the intervention is archived (Internet Archive, webcitation.org, and/or publishing the source code or screenshots/videos alongside the article). As pages behind login screens cannot be archived, consider creating demo pages which are accessible without login.

	1	2	3	4	5	
subitem not at all important	○	◉	○	○	○	essential

Rensa markering

Does your paper address subitem 5-vi?

Copy and paste relevant sections from the manuscript (include quotes in quotation marks "like this" to indicate direct quotes from your manuscript), or elaborate on this item by providing additional information not in the ms, or briefly explain why the item is not applicable/relevant for your study

Not currently included in manuscript. The service is currently running at alkoholhjalpen.se for logged in users, free of charge. Source code is available via the first author.

5-vii) Access

Access: Describe how participants accessed the application, in what setting/context, if they had to pay (or were paid) or not, whether they had to be a member of specific group. If known, describe how participants obtained "access to the platform and Internet" [1]. To ensure access for editors/reviewers /readers, consider to provide a "backdoor" login account or demo mode for reviewers/readers to explore the application (also important for archiving purposes, see vi).

	1	2	3	4	5	
subitem not at all important	○	○	○	◉	○	essential

Rensa markering

Ditt svar är för stort. Testa att förkorta en del av dina svar.

Does your paper address subitem 5-vii? *

Copy and paste relevant sections from the manuscript (include quotes in quotation marks "like this" to indicate direct quotes from your manuscript), or elaborate on this item by providing additional information not in the ms, or briefly explain why the item is not applicable/relevant for your study

"Participants were recruited at the Swedish internet site alkoholhjälpen.se, an open access website that provides information and a discussion forum to individuals seeking help online for alcohol consumption. The site has been publicly accessible since 2007. During the recruitment period for the current study, alkoholhjälpen.se had approximately 20 000 unique visitors every month and a mean of 100 new forum-posts every day. All service use was free of charge and no advertising was allowed on the website. "

5-viii) Mode of delivery, features/functionalities/components of the intervention and comparator, and the theoretical framework

Describe mode of delivery, features/functionalities/components of the intervention and comparator, and the theoretical framework [6] used to design them (instructional strategy [1], behaviour change techniques, persuasive features, etc., see e.g., [7, 8] for terminology). This includes an in-depth description of the content (including where it is coming from and who developed it) [1],” whether [and how] it is tailored to individual circumstances and allows users to track their progress and receive feedback” [6]. This also includes a description of communication delivery channels and – if computer-mediated communication is a component – whether communication was synchronous or asynchronous [6]. It also includes information on presentation strategies [1], including page design principles, average amount of text on pages, presence of hyperlinks to other resources, etc. [1].

	1	2	3	4	5	
subitem not at all important	○	○	○	◉	○	essential

Rensa markering

Ditt svar är för stort. Testa att förkorta en del av dina svar.

Does your paper address subitem 5-viii? *

Copy and paste relevant sections from the manuscript (include quotes in quotation marks "like this" to indicate direct quotes from your manuscript), or elaborate on this item by providing additional information not in the ms, or briefly explain why the item is not applicable/relevant for your study

Also see 5-ii.

"The program was based on self-help material used in previous studies on the internet and in specialist care [24,34,39,40]. Content and exercises in the program was based on motivational interviewing [35,36], relapse-prevention [37,38] and behavioural-self-control [39,40]. The program was divided into five main modules, three extra problem-solving modules and 10 fact sheets (see list of modules in Table 5). The length of the program was approximately 17000 words in total, with 5500 words in the extra modules and 3000 words in the fact sheets. See Figure 1 for example pictures of the program. Automatic reminders with suggestions on what module to work on were sent at 1-4, 6 and 8 weeks. Users were also encouraged to register alcohol-consumption or craving, and details on the situation where they drank or experienced craving. This was done in a private drinking-calendar"

"Participants in the therapist-guided ICBT group had the same access to the same program as the self-help ICBT described above. In addition to the program the therapist-guided ICBT group could communicate with a therapist, through asynchronous text-messages on the intervention website, during the 12 weeks of the program."

The control group was given access to a text-only information on changing their alcohol habits based on the text Alcohol and you"

5-ix) Describe use parameters

Describe use parameters (e.g., intended “doses” and optimal timing for use). Clarify what instructions or recommendations were given to the user, e.g., regarding timing, frequency, heaviness of use, if any, or was the intervention used ad libitum.

	1	2	3	4	5	
subitem not at all important	○	○	◉	○	○	essential

Rensa markering

Ditt svar är för stort. Testa att förkorta en del av dina svar.

Does your paper address subitem 5-ix?

Copy and paste relevant sections from the manuscript (include quotes in quotation marks "like this" to indicate direct quotes from your manuscript), or elaborate on this item by providing additional information not in the ms, or briefly explain why the item is not applicable/relevant for your study

"Automatic reminders with suggestions on what module to work on were sent at 1-4, 6 and 8 weeks. "

"To allow participant to complete the modules at the pace recommended, with room for some delay, the participants had access to the program during 12 weeks after allocation."

"Participants that completed 4 or more modules in the program was regarded as treatment completers. "

5-x) Clarify the level of human involvement

Clarify the level of human involvement (care providers or health professionals, also technical assistance) in the e-intervention or as co-intervention (detail number and expertise of professionals involved, if any, as well as “type of assistance offered, the timing and frequency of the support, how it is initiated, and the medium by which the assistance is delivered”. It may be necessary to distinguish between the level of human involvement required for the trial, and the level of human involvement required for a routine application outside of a RCT setting (discuss under item 21 – generalizability).

	1	2	3	4	5	
subitem not at all important	○	○	○	◉	○	essential

Rensa markering

Ditt svar är för stort. Testa att förkorta en del av dina svar.

Does your paper address subitem 5-x?

Copy and paste relevant sections from the manuscript (include quotes in quotation marks "like this" to indicate direct quotes from your manuscript), or elaborate on this item by providing additional information not in the ms, or briefly explain why the item is not applicable/relevant for your study

"In addition to the program the therapist-guided ICBT group could communicate with a therapist, through asynchronous text-messages on the intervention website, during the 12 weeks of the program. The support from the therapist was focused on motivating the user to continue to use the program and change their drinking. Each time the participant had completed any of the modules, the therapist wrote personal feedback and answered any questions about the program, via private comments on the web-platform. The feedback highlighted parts of what the participant had stated in the exercises included in each module, that were important from a MI or CBT perspective. Users that did not use or stopped using the program for several weeks were reminded by the therapist two times, with personal messages on the website (with notification on mail). "

"All three groups also had access to the discussion forum at the website"

"Participants who did not respond to this initial request received up to five automated e-mail reminders, a manual e-mail reminder and a mobile text message."

5-xi) Report any prompts/reminders used

Report any prompts/reminders used: Clarify if there were prompts (letters, emails, phone calls, SMS) to use the application, what triggered them, frequency etc. It may be necessary to distinguish between the level of prompts/reminders required for the trial, and the level of prompts/reminders for a routine application outside of a RCT setting (discuss under item 21 – generalizability).

	1	2	3	4	5	
subitem not at all important	○	○	◉	○	○	essential

Rensa markering

Ditt svar är för stort. Testa att förkorta en del av dina svar.

Does your paper address subitem 5-xi? *

Copy and paste relevant sections from the manuscript (include quotes in quotation marks "like this" to indicate direct quotes from your manuscript), or elaborate on this item by providing additional information not in the ms, or briefly explain why the item is not applicable/relevant for your study

"Automatic reminders with suggestions on what module to work on were sent at 1-4, 6 and 8 weeks. "

"Users that did not use or stopped using the program for several weeks were reminded by the therapist two times, with personal messages on the website (with notification on mail). "

"Participants who did not respond to this initial request received up to five automated e-mail reminders, a manual e-mail reminder and a mobile text message."

5-xii) Describe any co-interventions (incl. training/support)

Describe any co-interventions (incl. training/support): Clearly state any interventions that are provided in addition to the targeted eHealth intervention, as ehealth intervention may not be designed as stand-alone intervention. This includes training sessions and support [1]. It may be necessary to distinguish between the level of training required for the trial, and the level of training for a routine application outside of a RCT setting (discuss under item 21 – generalizability.

	1	2	3	4	5	
subitem not at all important	○	○	○	○	○	essential

Does your paper address subitem 5-xii? *

Copy and paste relevant sections from the manuscript (include quotes in quotation marks "like this" to indicate direct quotes from your manuscript), or elaborate on this item by providing additional information not in the ms, or briefly explain why the item is not applicable/relevant for your study

"All three groups also had access to the discussion forum at the website as well as facts on alcohol and health and information about how to find additional support within the health care or social welfare system. Communication between the server hosting the intervention and the participant was encrypted and protected with an individual login name and a password."

Ditt svar är för stort. Testa att förkorta en del av dina svar.

6a) Completely defined pre-specified primary and secondary outcome measures, including how and when they were assessed

Does your paper address CONSORT subitem 6a? *

Copy and paste relevant sections from the manuscript (include quotes in quotation marks "like this" to indicate direct quotes from your manuscript), or elaborate on this item by providing additional information not in the ms, or briefly explain why the item is not applicable/relevant for your study

"The primary outcome was the difference between groups in alcohol consumption, in mean weekly standard drinks. The number of standard drinks each of the seven days in the preceding week was self-reported using the Timeline Follow-back (TLFB) method [48]. One standard drink contains 12 grams of pure alcohol according to the Swedish definition."

"Alcohol-related outcomes were assessed with a number of different instruments. The AUDIT [38] is a 10-item instrument, covering both alcohol-consumption and problems, that has been validated in Swedish and via computer [51,52]. The AUDIT total score was used as a continuous measure of alcohol related problems."

"Alcohol dependence was assessed by the number of self-reported alcohol dependence criteria during the last year according to ICD-10 [4]. Alcohol Use Disorder was assessed by the number of self-rated of alcohol use disorder criteria during the last year according to DSM-5 [54]. "

"Health related quality of life was assessed with EuroQol-5 dimension (EQ-5D-5L)... Symptoms of depression were measured by the total score of Montgomery Asberg Depression Rating Scale – Self Rated (MADRS-S) [51,52]. Symptoms of anxiety were measured by the total score of Generalized Anxiety Disorder Assessment–7 Items (GAD-7; [53,54])."

"Follow-up was conducted 3 and 6 months after recruitment. "

"The same questionnaires with all primary and secondary outcomes used at baseline were also used at follow-up, adjusted for the time since last assessment (3 months)."

Ditt svar är för stort. Testa att förkorta en del av dina svar.

6a-i) Online questionnaires: describe if they were validated for online use and apply CHERRIES items to describe how the questionnaires were designed/deployed

If outcomes were obtained through online questionnaires, describe if they were validated for online use and apply CHERRIES items to describe how the questionnaires were designed/deployed [9].

	1	2	3	4	5	
subitem not at all important	○	○	●	○	○	essential

Rensa markering

Does your paper address subitem 6a-i?

Copy and paste relevant sections from manuscript text

"The TLFB has been shown to be a valid and reliable procedure to document recent drinking also when administered via internet"

"The AUDIT [38] is a 10-item instrument, covering both alcohol-consumption and problems, that has been validated in Swedish and via computer [51,52]. "

6a-ii) Describe whether and how "use" (including intensity of use/dosage) was defined/measured/monitored

Describe whether and how "use" (including intensity of use/dosage) was defined/measured/monitored (logins, logfile analysis, etc.). Use/adoption metrics are important process outcomes that should be reported in any ehealth trial.

	1	2	3	4	5	
subitem not at all important	○	○	○	●	○	essential

Rensa markering

Ditt svar är för stort. Testa att förkorta en del av dina svar.

Does your paper address subitem 6a-ii?

Copy and paste relevant sections from manuscript text

"All use of the intervention was logged for each user. Participants that completed 4 or more modules in the program was regarded as treatment completers."

6a-iii) Describe whether, how, and when qualitative feedback from participants was obtained

Describe whether, how, and when qualitative feedback from participants was obtained (e.g., through emails, feedback forms, interviews, focus groups).

	1	2	3	4	5	
subitem not at all important	○	○	◉	○	○	essential

Rensa markering

Does your paper address subitem 6a-iii?

Copy and paste relevant sections from manuscript text

"Working alliance was measured 3 and 6 weeks after randomization...
Session Rating Scale (SRS) [62] in regard to using the website and the intervention that they had received. After each module in the program users in the Therapist-guided ICBT and Self-help ICBT groups could rate how helpful they found the module"

"The baseline questionnaires were followed by a survey on why participants choose to use web-based services and their preferences regarding such services."

6b) Any changes to trial outcomes after the trial commenced, with reasons

Ditt svar är för stort. Testa att förkorta en del av dina svar.

Does your paper address CONSORT subitem 6b? *

Copy and paste relevant sections from the manuscript (include quotes in quotation marks "like this" to indicate direct quotes from your manuscript), or elaborate on this item by providing additional information not in the ms, or briefly explain why the item is not applicable/relevant for your study

No changes to trial outcomes were made after the trial started.

7a) How sample size was determined

NPT: When applicable, details of whether and how the clustering by care provides or centers was addressed

7a-i) Describe whether and how expected attrition was taken into account when calculating the sample size

Describe whether and how expected attrition was taken into account when calculating the sample size.

	1	2	3	4	5	
subitem not at all important	○	○	◉	○	○	essential

Rensa markering

Does your paper address subitem 7a-i?

Copy and paste relevant sections from manuscript title (include quotes in quotation marks "like this" to indicate direct quotes from your manuscript), or elaborate on this item by providing additional information not in the ms, or briefly explain why the item is not applicable/relevant for your study

"With α = 0.05 and 80 percent power, n=394 per group was required to the desired effect size, totaling n=1182. To allow analyses also on observed data only, assuming 50% missing data at follow-up, the enrollment goal was however increased to n=2400. "

7b) When applicable, explanation of any interim analyses and stopping guidelines

Ditt svar är för stort. Testa att förkorta en del av dina svar.

Does your paper address CONSORT subitem 7b? *

Copy and paste relevant sections from the manuscript (include quotes in quotation marks "like this" to indicate direct quotes from your manuscript), or elaborate on this item by providing additional information not in the ms, or briefly explain why the item is not applicable/relevant for your study

We did not use any interim analyses or stopping guidelines. For funding reasons the maximum recruitment window was pre-specified to 24 months.

8a) Method used to generate the random allocation sequence

NPT: When applicable, how care providers were allocated to each trial group

Does your paper address CONSORT subitem 8a? *

Copy and paste relevant sections from the manuscript (include quotes in quotation marks "like this" to indicate direct quotes from your manuscript), or elaborate on this item by providing additional information not in the ms, or briefly explain why the item is not applicable/relevant for your study

"Participants who completed the baseline measures were randomized according to a fully automated and concealed procedure in the online platform."

8b) Type of randomisation; details of any restriction (such as blocking and block size)

Does your paper address CONSORT subitem 8b? *

Copy and paste relevant sections from the manuscript (include quotes in quotation marks "like this" to indicate direct quotes from your manuscript), or elaborate on this item by providing additional information not in the ms, or briefly explain why the item is not applicable/relevant for your study

"ratio of 1:1:1 and block size of 30"

Ditt svar är för stort. Testa att förkorta en del av dina svar.

9) Mechanism used to implement the random allocation sequence (such as sequentially numbered containers), describing any steps taken to conceal the sequence until interventions were assigned

Does your paper address CONSORT subitem 9? *

Copy and paste relevant sections from the manuscript (include quotes in quotation marks "like this" to indicate direct quotes from your manuscript), or elaborate on this item by providing additional information not in the ms, or briefly explain why the item is not applicable/relevant for your study

"Participants who completed the baseline measures were randomized according to a fully automated and concealed procedure in the online platform."

Both the recruitment and the randomization and allocation were fully automated, pre-programmed and concealed within the platform. None of the researchers or therapists had any contact with the users before they were randomized. And no one had knowledge of at what user the program switched to the next block, e.g. the size of the initial block when the recruitment started.

10) Who generated the random allocation sequence, who enrolled participants, and who assigned participants to interventions

Does your paper address CONSORT subitem 10? *

Copy and paste relevant sections from the manuscript (include quotes in quotation marks "like this" to indicate direct quotes from your manuscript), or elaborate on this item by providing additional information not in the ms, or briefly explain why the item is not applicable/relevant for your study

Both the recruitment and the randomization and allocation were fully automated and pre-programmed by the firste author.

11a) If done, who was blinded after assignment to interventions (for example, participants, care providers, those assessing outcomes) and how

NPT: Whether or not administering co-interventions were blinded to group assignment

Ditt svar är för stort. Testa att förkorta en del av dina svar.

11a-i) Specify who was blinded, and who wasn't

Specify who was blinded, and who wasn't. Usually, in web-based trials it is not possible to blind the participants [1, 3] (this should be clearly acknowledged), but it may be possible to blind outcome assessors, those doing data analysis or those administering co-interventions (if any).

	1	2	3	4	5	
subitem not at all important	○	○	○	◉	○	essential

Rensa markering

Does your paper address subitem 11a-i? *

Copy and paste relevant sections from the manuscript (include quotes in quotation marks "like this" to indicate direct quotes from your manuscript), or elaborate on this item by providing additional information not in the ms, or briefly explain why the item is not applicable/relevant for your study

"Participants were blinded to what kind of support that participants in the other groups received."

11a-ii) Discuss e.g., whether participants knew which intervention was the "intervention of interest" and which one was the "comparator"

Informed consent procedures (4a-ii) can create biases and certain expectations - discuss e.g., whether participants knew which intervention was the "intervention of interest" and which one was the "comparator".

	1	2	3	4	5	
subitem not at all important	○	○	◉	○	○	essential

Rensa markering

Ditt svar är för stort. Testa att förkorta en del av dina svar.

Does your paper address subitem 11a-ii?

Copy and paste relevant sections from the manuscript (include quotes in quotation marks "like this" to indicate direct quotes from your manuscript), or elaborate on this item by providing additional information not in the ms, or briefly explain why the item is not applicable/relevant for your study

"Participants were blinded to what kind of support that participants in the other groups received. "

"Interested users were informed that they would answer a survey and be randomized to one of three different forms of support, but were not informed about the specifics."

11b) If relevant, description of the similarity of interventions

(this item is usually not relevant for ehealth trials as it refers to similarity of a placebo or sham intervention to a active medication/intervention)

Does your paper address CONSORT subitem 11b? *

Copy and paste relevant sections from the manuscript (include quotes in quotation marks "like this" to indicate direct quotes from your manuscript), or elaborate on this item by providing additional information not in the ms, or briefly explain why the item is not applicable/relevant for your study

"Participants in the therapist-guided ICBT group had the same access to the same program as the self-help ICBT described above."

"All three groups also had access to the discussion forum at the website as well as facts on alcohol and health and information about how to find additional support within the health care or social welfare system. "

12a) Statistical methods used to compare groups for primary and secondary outcomes

NPT: When applicable, details of whether and how the clustering by care providers or centers was addressed

Ditt svar är för stort. Testa att förkorta en del av dina svar.

Does your paper address CONSORT subitem 12a? *

Copy and paste relevant sections from the manuscript (include quotes in quotation marks "like this" to indicate direct quotes from your manuscript), or elaborate on this item by providing additional information not in the ms, or briefly explain why the item is not applicable/relevant for your study

"All statistical analyses were two-sided tests and, unless specified otherwise, used a significance level of p < .05. Factorial ANOVAs were used to test differences in baseline measures between users who were retained and users who were lost to follow-up, including interactions between group and lost to follow-up (at either 3 month or 6 month)."

"In accordance with the original protocol, differences in observed means at each follow-up were analyzed with t-tests, under the missing at random assumption; significant contrasts were however supplemented with tipping point sensitivity analyses that systematically imputed missing data at a group-level across a range of plausible mean values in the two non-respondent groups (with the same standard deviation) [65,66]."

12a-i) Imputation techniques to deal with attrition / missing values

Imputation techniques to deal with attrition / missing values: Not all participants will use the intervention/comparator as intended and attrition is typically high in ehealth trials. Specify how participants who did not use the application or dropped out from the trial were treated in the statistical analysis (a complete case analysis is strongly discouraged, and simple imputation techniques such as LOCF may also be problematic [4]).

	1	2	3	4	5	
subitem not at all important	○	○	◉	○	○	essential

Rensa markering

Does your paper address subitem 12a-i? *

Copy and paste relevant sections from the manuscript (include quotes in quotation marks "like this" to indicate direct quotes from your manuscript), or elaborate on this item by providing additional information not in the ms, or briefly explain why the item is not applicable/relevant for your study

"mixed effects modelling that would be fully compliant with the intention to treat principle, and better equipped to handle the presumed high degree of missing data"

Ditt svar är för stort. Testa att förkorta en del av dina svar.

12b) Methods for additional analyses, such as subgroup analyses and adjusted analyses

Does your paper address CONSORT subitem 12b? *

Copy and paste relevant sections from the manuscript (include quotes in quotation marks "like this" to indicate direct quotes from your manuscript), or elaborate on this item by providing additional information not in the ms, or briefly explain why the item is not applicable/relevant for your study

"Prior to analysing the data, the decision was made to supplement the original analytic protocol with mixed effects modelling that would be fully compliant with the intention to treat principle, and better equipped to handle the presumed high degree of missing data [63]. By modelling data at both group (fixed) and individual (random) levels, mixed models are well-suited for data from repeated observations (modeling clustering of data at an individual level) [67] and maximum likelihood estimation is used to handle missing data [68]. Analyzing outcomes with (generalized) mixed effects models also allowed the use of family functions more appropriate for the distribution of the outcome. "

X26) REB/IRB Approval and Ethical Considerations [recommended as subheading under "Methods"] (not a CONSORT item)

X26-i) Comment on ethics committee approval

	1	2	3	4	5	
subitem not at all important	○	○	○	◉	○	essential

Rensa markering

Ditt svar är för stort. Testa att förkorta en del av dina svar.

Does your paper address subitem X26-i?

Copy and paste relevant sections from the manuscript (include quotes in quotation marks "like this" to indicate direct quotes from your manuscript), or elaborate on this item by providing additional information not in the ms, or briefly explain why the item is not applicable/relevant for your study

"This trial was approved by the Stockholm Regional Ethical Review Board (No. 2014/1758-31/2)."

x26-ii) Outline informed consent procedures

Outline informed consent procedures e.g., if consent was obtained offline or online (how? Checkbox, etc.?), and what information was provided (see 4a-ii). See [6] for some items to be included in informed consent documents.

	1	2	3	4	5	
subitem not at all important	○	◉	○	○	○	essential

Rensa markering

Does your paper address subitem X26-ii?

Copy and paste relevant sections from the manuscript (include quotes in quotation marks "like this" to indicate direct quotes from your manuscript), or elaborate on this item by providing additional information not in the ms, or briefly explain why the item is not applicable/relevant for your study

"They were then directed to a screening page where they were required to give informed consent for participation in the study, answer demographic questions, the questions in the Alcohol Use Disorder Identification Test (AUDIT) [38] and questions about alcohol dependence (ICD-10) criteria."

Obtained online with a Checkbox.

Ditt svar är för stort. Testa att förkorta en del av dina svar.

X26-iii) Safety and security procedures

Safety and security procedures, incl. privacy considerations, and any steps taken to reduce the likelihood or detection of harm (e.g., education and training, availability of a hotline)

	1	2	3	4	5	
subitem not at all important	○	○	●	○	○	essential

Rensa markering

Does your paper address subitem X26-iii?

Copy and paste relevant sections from the manuscript (include quotes in quotation marks "like this" to indicate direct quotes from your manuscript), or elaborate on this item by providing additional information not in the ms, or briefly explain why the item is not applicable/relevant for your study

"Communication between the server hosting the intervention and the participant was encrypted and protected with an individual login name and a password."

"email-address and a mobile phone number for notifications and follow-up reminders. Email and phone numbers were not verified, nor used for identification or for any other purposes."

RESULTS

13a) For each group, the numbers of participants who were randomly assigned, received intended treatment, and were analysed for the primary outcome

NPT: The number of care providers or centers performing the intervention in each group and the number of patients treated by each care provider in each center

Ditt svar är för stort. Testa att förkorta en del av dina svar.

Does your paper address CONSORT subitem 13a? *

Copy and paste relevant sections from the manuscript (include quotes in quotation marks "like this" to indicate direct quotes from your manuscript), or elaborate on this item by providing additional information not in the ms, or briefly explain why the item is not applicable/relevant for your study

"A total of 1169 participants were randomly allocated to the three study arms (see Figure 2 for flow chart)."

13b) For each group, losses and exclusions after randomisation, together with reasons

Ditt svar är för stort. Testa att förkorta en del av dina svar.

Does your paper address CONSORT subitem 13b? (NOTE: Preferably, this is shown in a CONSORT flow diagram) *

Copy and paste relevant sections from the manuscript (include quotes in quotation marks "like this" to indicate direct quotes from your manuscript), or elaborate on this item by providing additional information not in the ms, or briefly explain why the item is not applicable/relevant for your study

In CONSORT flow:

"Allocated to therapist-guided ICBT (n=386)*
- Did not start the program (n=101) 26%
- Did not log in again** (n=53) 14%
3-month Follow-Up
Lost to follow-up (n=186) 48%
- No response (n=178)
- Declined to participate (n=22)
6-month Follow-Up
Lost to follow up (n=237) 60%
- No response (n=209)
- Declined to participate (n=28)"

"Allocated to self-help ICBT (n=391)
- Did not start the program (n=113) 29%
- Did not log in again** (n=83) 21%
3-month Follow-Up
Lost to follow-up (n=208) 53%
- No response (n=196)
- Declined to participate (n=12)
6-month Follow-Up
Lost to follow up (n=217) 56%
- No response (n=202)
- Declined to participate (n=15)"

"Allocated to control (n=392)
- Did not log in again** (n=91) 23%
3-month Follow-Up
Lost to follow-up (n=173) 44%
- No response (n=156)
- Declined to participate (n=17)
6-month Follow-Up
Lost to follow up (n=206) 53%
- No response (n=181)
- Declined to participate (n=25)"

Ditt svar är för stort. Testa att förkorta en del av dina svar.

13b-i) Attrition diagram

Strongly recommended: An attrition diagram (e.g., proportion of participants still logging in or using the intervention/comparator in each group plotted over time, similar to a survival curve) or other figures or tables demonstrating usage/dose/engagement.

	1	2	3	4	5	
subitem not at all important	○	○	◉	○	○	essential

Rensa markering

Does your paper address subitem 13b-i?

Copy and paste relevant sections from the manuscript or cite the figure number if applicable (include quotes in quotation marks "like this" to indicate direct quotes from your manuscript), or elaborate on this item by providing additional information not in the ms, or briefly explain why the item is not applicable/relevant for your study

"Table 5 for detailed program use."

14a) Dates defining the periods of recruitment and follow-up

Does your paper address CONSORT subitem 14a? *

Copy and paste relevant sections from the manuscript (include quotes in quotation marks "like this" to indicate direct quotes from your manuscript), or elaborate on this item by providing additional information not in the ms, or briefly explain why the item is not applicable/relevant for your study

"the recruitment period, from March 2015 to March 2017"

"Follow-up was conducted 3 and 6 months after recruitment."

"Additional follow-ups at 12 and 24 months after recruitment"

Ditt svar är för stort. Testa att förkorta en del av dina svar.

14a-i) Indicate if critical “secular events” fell into the study period

Indicate if critical “secular events” fell into the study period, e.g., significant changes in Internet resources available or “changes in computer hardware or Internet delivery resources”

	1	2	3	4	5	
subitem not at all important	○	◉	○	○	○	essential

Rensa markering

Does your paper address subitem 14a-i?

Copy and paste relevant sections from the manuscript (include quotes in quotation marks "like this" to indicate direct quotes from your manuscript), or elaborate on this item by providing additional information not in the ms, or briefly explain why the item is not applicable/relevant for your study

No such event occured during the study period.

14b) Why the trial ended or was stopped (early)

Does your paper address CONSORT subitem 14b? *

Copy and paste relevant sections from the manuscript (include quotes in quotation marks "like this" to indicate direct quotes from your manuscript), or elaborate on this item by providing additional information not in the ms, or briefly explain why the item is not applicable/relevant for your study

"This was lower than the target sample size required to adjust analyses on observed data only for estimated attrition at follow-up, but only negligibly smaller than in the raw power calculation with estimated missing data (n=1182). Recruitment nonetheless ceased after the pre-specified 24 months recruitment window, for funding reasons."

15) A table showing baseline demographic and clinical characteristics for each group

[illegible]

Ditt svar är för stort. Testa att förkorta en del av dina svar.

Does your paper address CONSORT subitem 15? *

Copy and paste relevant sections from the manuscript (include quotes in quotation marks "like this" to indicate direct quotes from your manuscript), or elaborate on this item by providing additional information not in the ms, or briefly explain why the item is not applicable/relevant for your study

"Full demographic and clinical variables at baseline are presented in Table 1."

15-i) Report demographics associated with digital divide issues

In ehealth trials it is particularly important to report demographics associated with digital divide issues, such as age, education, gender, social-economic status, computer/Internet/ehealth literacy of the participants, if known.

	1	2	3	4	5	
subitem not at all important	○	◉	○	○	○	essential

Rensa markering

Does your paper address subitem 15-i? *

Copy and paste relevant sections from the manuscript (include quotes in quotation marks "like this" to indicate direct quotes from your manuscript), or elaborate on this item by providing additional information not in the ms, or briefly explain why the item is not applicable/relevant for your study

Age, education, gender, employment and housing are reported in table 1.

16) For each group, number of participants (denominator) included in each analysis and whether the analysis was by original assigned groups

Ditt svar är för stort. Testa att förkorta en del av dina svar.

16-i) Report multiple “denominators” and provide definitions

Report multiple “denominators” and provide definitions: Report N’s (and effect sizes) “across a range of study participation [and use] thresholds” [1], e.g., N exposed, N consented, N used more than x times, N used more than y weeks, N participants “used” the intervention/comparator at specific pre-defined time points of interest (in absolute and relative numbers per group). Always clearly define “use” of the intervention.

	1	2	3	4	5	
subitem not at all important	○	○	◉	○	○	essential

Rensa markering

Does your paper address subitem 16-i? *

Copy and paste relevant sections from the manuscript (include quotes in quotation marks "like this" to indicate direct quotes from your manuscript), or elaborate on this item by providing additional information not in the ms, or briefly explain why the item is not applicable/relevant for your study

In CONSORT flow, see 13b.

16-ii) Primary analysis should be intent-to-treat

Primary analysis should be intent-to-treat, secondary analyses could include comparing only “users”, with the appropriate caveats that this is no longer a randomized sample (see 18-i).

	1	2	3	4	5	
subitem not at all important	○	○	○	◉	○	essential

Rensa markering

Ditt svar är för stort. Testa att förkorta en del av dina svar.

Does your paper address subitem 16-ii?

Copy and paste relevant sections from the manuscript (include quotes in quotation marks "like this" to indicate direct quotes from your manuscript), or elaborate on this item by providing additional information not in the ms, or briefly explain why the item is not applicable/relevant for your study

"In accordance with the original protocol, differences in observed means at each follow-up were analyzed with t-tests, under the missing at random assumption; significant contrasts were however supplemented with tipping point sensitivity analyses that systematically imputed missing data at a group-level across a range of plausible mean values in the two non-respondent groups (with the same standard deviation) [65,66]."

"Prior to analysing the data, the decision was made to supplement the original analytic protocol with mixed effects modelling that would be fully compliant with the intention to treat principle"

17a) For each primary and secondary outcome, results for each group, and the estimated effect size and its precision (such as 95% confidence interval)

Ditt svar är för stort. Testa att förkorta en del av dina svar.

Does your paper address CONSORT subitem 17a? *

Copy and paste relevant sections from the manuscript (include quotes in quotation marks "like this" to indicate direct quotes from your manuscript), or elaborate on this item by providing additional information not in the ms, or briefly explain why the item is not applicable/relevant for your study

"The therapist-guided ICBT group had significantly lower mean weekly alcohol consumption compared to the control group at 3 months (difference=-3.84, 95 CI=-6.53 to -1.16, d=0.27)."

"No significant differences in weekly alcohol consumption were found between the self-help ICBT group and the control group (difference=-2.41, 95 CI=-5.53 to 0.71) or between the therapist-guided ICBT group and the self-help ICBT group (difference =-1.43, 95 CI -4.26 to 1.40)"

"At 3 months (post-treatment) there were significant differences between the therapist-guided ICBT group and the control group in the secondary outcomes AUDIT (difference=-2.91, 95 CI=-4.33 to-1.50, d=0.39), AUDIT-C (difference=-0.77, CI=-1.34 to -.20, d=0.26), DSM-5 (difference=-0.76, CI= -1.34 to -.17, d=0.25) and ICD-10 (difference=-0.47, 95 CI=-0.82 to -0.14, d=0.26). A difference was also found between the self-help ICBT and control group on the AUDIT (difference=-1.95, 95 CI=-3.44 to -.46, d=0.26) at 3 months. No significant differences in secondary outcomes between the therapist-guided ICBT and self-help ICBT were found at 3 months. "

"There were no significant differences in weekly alcohol consumption between any of the groups at 6 months. The difference between the therapist-guided ICBT and control group was -0.60 (95 CI=-3.70 to 2.50), between self-help ICBT and control group -0.45 (95 CI=-3.87 to 2.96) and between the therapist-guided ICBT and the self-help ICBT -0.15 (95 CI=-3.70 to 3.41). No significant differences in secondary outcomes between any of the groups were found at 6 months. See Table 3 for details and supplementary materials Table S2 for tipping point analyses of secondary outcomes."

Ditt svar är för stort. Testa att förkorta en del av dina svar.

17a-i) Presentation of process outcomes such as metrics of use and intensity of use

In addition to primary/secondary (clinical) outcomes, the presentation of process outcomes such as metrics of use and intensity of use (dose, exposure) and their operational definitions is critical. This does not only refer to metrics of attrition (13-b) (often a binary variable), but also to more continuous exposure metrics such as “average session length”. These must be accompanied by a technical description how a metric like a “session” is defined (e.g., timeout after idle time) [1] (report under item 6a).

	1	2	3	4	5	
subitem not at all important	○	○	○	◉	○	essential

Rensa markering

Does your paper address subitem 17a-i?

Copy and paste relevant sections from the manuscript (include quotes in quotation marks "like this" to indicate direct quotes from your manuscript), or elaborate on this item by providing additional information not in the ms, or briefly explain why the item is not applicable/relevant for your study

"The number of modules completed by the therapist-guided ICBT (M=3.3 (SD=3.5)) was significantly higher (t= 2.9, p=.004) compared to the self-help ICBT (M=2.6 (SD=3.2)) but there was no significant difference in number of calendar entries (therapist-guided ICBT: M=39 (SD=61) and self-help ICBT M=37 (SD=102); t=0.60, p=.58). In the therapist-guided ICBT 40% were treatment completers and in the self-help ICBT 30% (Chi2=6.46, p=.011). In the therapist-guided ICBT 58% sent at least one message to their therapists. They sent a mean of 4.7 (SD=4.7) messages and received a mean of 6.0 (SD=4.1) from their therapist. See Table 5 for detailed program use. The number of participants who used the discussion forum was higher in the control group compared to the therapist-guided ICBT group (n=107 (27%) vs n=71 (18%); Chi2=8.74, p=.003). But there were no significant differences between self-help ICBT (n=88, 22%) and the therapist-guided ICBT or the control group in forum usage. "

17b) For binary outcomes, presentation of both absolute and relative effect sizes is recommended

Ditt svar är för stort. Testa att förkorta en del av dina svar.

Does your paper address CONSORT subitem 17b? *

Copy and paste relevant sections from the manuscript (include quotes in quotation marks "like this" to indicate direct quotes from your manuscript), or elaborate on this item by providing additional information not in the ms, or briefly explain why the item is not applicable/relevant for your study

Not relevant for the study.

18) Results of any other analyses performed, including subgroup analyses and adjusted analyses, distinguishing pre-specified from exploratory

Does your paper address CONSORT subitem 18? *

Copy and paste relevant sections from the manuscript (include quotes in quotation marks "like this" to indicate direct quotes from your manuscript), or elaborate on this item by providing additional information not in the ms, or briefly explain why the item is not applicable/relevant for your study

"The mixed model ITT analysis showed a significant larger decrease in weekly alcohol consumption over time in the therapist-guided ICBT (time x therapist: t=-2.33, p=.02) compared to control, but not in the self-help ICBT (time x self-help: t=-1.68, p=.09) compared to control (see models in table 4). A model comparing only the participants in the therapist-guided ICBT and self-help ICBT did not reveal any significant effect of group x time (t=-0.57, p=.57) There was a significant decrease in weekly alcohol consumption over time for participants in all three groups according to the estimate of time in the unconditional model (t=-11.98, p>.001)."

"According to the mixed model analysis of secondary outcomes a significantly larger decrease in AUDIT over time was found for both the therapist-guided ICBT and self-help ICBT compared a to control as shown by therapist-guided ICBT x time (t=-3.55, p<.001) and self-help ICBT x time (-2.23, p=.026). There were also significant therapist-guided ICBT x time group effects in AUDIT-C (-2.71; p=.007), ICD (-1.89, p=.06) and DSM-5 (-2.10, p=.036). No other significant time x group effects were found in the mixed model analysis of secondary outcomes. Over time there was significant decrease among all participants on the AUDIT, AUDIT-C, ICD, DSM-5, MADRS-S, GAD-7, binge-drinking days and drinks on drinking days as well significant increase inEQ-5D-5L and non-drinking days. See supplementary material Table S3 for additional models."

Ditt svar är för stort. Testa att förkorta en del av dina svar.

18-i) Subgroup analysis of comparing only users

A subgroup analysis of comparing only users is not uncommon in ehealth trials, but if done, it must be stressed that this is a self-selected sample and no longer an unbiased sample from a randomized trial (see 16-iii).

	1	2	3	4	5	
subitem not at all important	○	◉	○	○	○	essential

Rensa markering

Does your paper address subitem 18-i?

Copy and paste relevant sections from the manuscript (include quotes in quotation marks "like this" to indicate direct quotes from your manuscript), or elaborate on this item by providing additional information not in the ms, or briefly explain why the item is not applicable/relevant for your study

Not applicable in this study.

19) All important harms or unintended effects in each group

(for specific guidance see CONSORT for harms)

Does your paper address CONSORT subitem 19? *

Copy and paste relevant sections from the manuscript (include quotes in quotation marks "like this" to indicate direct quotes from your manuscript), or elaborate on this item by providing additional information not in the ms, or briefly explain why the item is not applicable/relevant for your study

"There were no reported adverse events due to the intervention reported by participants. Among follow-up participants at 6 months 17 of 508 (3%) had changed their alcohol use to a more severe category according to AUDIT and 99 of 508 (19%) stayed within the highest AUDIT category."

Ditt svar är för stort. Testa att förkorta en del av dina svar.

19-i) Include privacy breaches, technical problems

Include privacy breaches, technical problems. This does not only include physical “harm” to participants, but also incidents such as perceived or real privacy breaches [1], technical problems, and other unexpected/unintended incidents. “Unintended effects” also includes unintended positive effects [2].

	1	2	3	4	5	
subitem not at all important	○	○	◉	○	○	essential

Rensa markering

Does your paper address subitem 19-i?

Copy and paste relevant sections from the manuscript (include quotes in quotation marks "like this" to indicate direct quotes from your manuscript), or elaborate on this item by providing additional information not in the ms, or briefly explain why the item is not applicable/relevant for your study

No such events were reported by users in this study.

19-ii) Include qualitative feedback from participants or observations from staff/researchers

Include qualitative feedback from participants or observations from staff/researchers, if available, on strengths and shortcomings of the application, especially if they point to unintended/unexpected effects or uses. This includes (if available) reasons for why people did or did not use the application as intended by the developers.

	1	2	3	4	5	
subitem not at all important	○	○	◉	○	○	essential

Rensa markering

Ditt svar är för stort. Testa att förkorta en del av dina svar.

Does your paper address subitem 19-ii?

Copy and paste relevant sections from the manuscript (include quotes in quotation marks "like this" to indicate direct quotes from your manuscript), or elaborate on this item by providing additional information not in the ms, or briefly explain why the item is not applicable/relevant for your study

"Data from this survey will be presented elsewhere."

DISCUSSION

22) Interpretation consistent with results, balancing benefits and harms, and considering other relevant evidence

NPT: In addition, take into account the choice of the comparator, lack of or partial blinding, and unequal expertise of care providers or centers in each group

22-i) Restate study questions and summarize the answers suggested by the data, starting with primary outcomes and process outcomes (use)

Restate study questions and summarize the answers suggested by the data, starting with primary outcomes and process outcomes (use).

	1	2	3	4	5	
subitem not at all important	○	○	○	◉	○	essential

Rensa markering

Ditt svar är för stort. Testa att förkorta en del av dina svar.

Does your paper address subitem 22-i? *

Copy and paste relevant sections from the manuscript (include quotes in quotation marks "like this" to indicate direct quotes from your manuscript), or elaborate on this item by providing additional information not in the ms, or briefly explain why the item is not applicable/relevant for your study

"The results only partly confirmed the first hypothesis. Participants randomized to therapist-guided ICBT reduced their weekly alcohol consumption as well as alcohol related problems (measured with the AUDIT), and signs of Alcohol Use Disorder, significantly more than participants in the control group at 3-months follow-up; they also reduced their alcohol related problems more than participants in the control group at 6-months follow-up. "

"The results did not confirm the second hypothesis. Self-help ICBT was not more effective than the control condition in changing alcohol consumption. This is in line with results from the first study of alkoholhjälpen.se [34] and two other large studies of publicly available services [26,31]. However, the self-help ICBT group did change their alcohol related problems significantly more compared to controls at 3 months. No support was found for the third hypothesis. There were no significant differences in changed drinking or other outcomes between therapist-guided ICBT and self-help ICBT. "

22-ii) Highlight unanswered new questions, suggest future research

Highlight unanswered new questions, suggest future research.

	1	2	3	4	5	
subitem not at all important	○	○	○	◉	○	essential

Rensa markering

Ditt svar är för stort. Testa att förkorta en del av dina svar.

Does your paper address subitem 22-ii?

Copy and paste relevant sections from the manuscript (include quotes in quotation marks "like this" to indicate direct quotes from your manuscript), or elaborate on this item by providing additional information not in the ms, or briefly explain why the item is not applicable/relevant for your study

"These findings are in line with the results of previous studies on therapist-guided ICBT but with smaller differences between the groups. "

"no significant differences in changed drinking or other outcomes between therapist-guided ICBT and self-help ICBT."

There is still a need for more studies on multi-session internet intervention for harmful alcohol use and alcohol dependence, including studies with long-term follow-up. Low-risk drinking was reported by 43% of participants at both follow-ups, but without differences between groups. Only some internet help-seekers might need ICBT and therapist guidance in order to change their drinking when they use internet interventions. Others, who did not improve, might have benefited from more intensive support. A model of support-on-demand or accelerated care could be tested in future studies on the internet. One important challenge for future studies is to improve follow-up rates as well as adherence to interventions without reducing the willingness to use the interventions. Increased demands on users to identify themselves or have contact with a professional might make people, who wish to stay anonymous or feel ashamed or stigmatized, more reluctant to seek support [82]. Treatment seeking increases the rates of recovery from alcohol dependence [83] and internet interventions seem to be a possible way to reach individuals currently not seeking treatment [84] but it is still unclear if internet interventions actually increase treatment seeking. Research on other psychiatric disorders [85] and on internet alcohol interventions so far [17] suggests that therapist-guided internet treatment have effects comparable to those of face-to-face treatment but more studies are needed that directly compare these interventions [86]. In sparsely populated countries such as Sweden, where some people have to travel far to visit a clinic in person, both psychological treatment [87], medical management [88] and after-care [89] could in part be handled with internet interventions. We need more studies to understand how internet interventions can be used effectively to improve treatment for people with alcohol dependence.

20) Trial limitations, addressing sources of potential bias, imprecision, and, if relevant, multiplicity of analyses

Ditt svar är för stort. Testa att förkorta en del av dina svar.

20-i) Typical limitations in ehealth trials

Typical limitations in ehealth trials: Participants in ehealth trials are rarely blinded. Ehealth trials often look at a multiplicity of outcomes, increasing risk for a Type I error. Discuss biases due to non-use of the intervention/usability issues, biases through informed consent procedures, unexpected events.

	1	2	3	4	5	
subitem not at all important	○	○	○	◉	○	essential

Rensa markering

Does your paper address subitem 20-i? *

Copy and paste relevant sections from the manuscript (include quotes in quotation marks "like this" to indicate direct quotes from your manuscript), or elaborate on this item by providing additional information not in the ms, or briefly explain why the item is not applicable/relevant for your study

"Despite great efforts in reminding and reinforcing participants for answering follow-up questions, attrition was high. Participants lost to follow-up showed some differences to those retained, a factor which limits the generalizability of the results. However, tipping point analyses and the fact that there were no significant arm × attrition interactions on outcomes, suggest that no sampling bias was introduced as result of the attrition. The attrition could be a consequence of allowing users to be relatively anonymous and having a fast and accessible way of signing up for the study, lowering the threshold for engagement. Loss to follow-up also entails that we were powered to detect a smaller than planned effect size. In the between-group comparison of the self-help and therapist-guided arms at the three-month follow-up, observed sample sizes would give 80% power to detect d>0.29, which is still to be considered a small difference. We can however not rule out that the true difference is smaller than this. Adherence to the program was relatively low, with only 30% completers in the self-help ICBT and 40% in the therapist-guided ICBT, which is consistent with previous studies on ICBT (see supplementary material Table S1). Higher adherence might improve the effects of the internet-based program. Due to the online setting the participants did not go through a clinical diagnostic interview and some participants may not have been diagnosed as having alcohol dependence had an interview been included in the study design. "

21) Generalisability (external validity, applicability) of the trial findings

NPT: External validity of the trial findings according to the intervention, comparators, patients, and care [illegible]

Ditt svar är för stort. Testa att förkorta en del av dina svar.

21-i) Generalizability to other populations

Generalizability to other populations: In particular, discuss generalizability to a general Internet population, outside of a RCT setting, and general patient population, including applicability of the study results for other organizations

	1	2	3	4	5	
subitem not at all important	○	○	○	◉	○	essential

Rensa markering

Does your paper address subitem 21-i?

Copy and paste relevant sections from the manuscript (include quotes in quotation marks "like this" to indicate direct quotes from your manuscript), or elaborate on this item by providing additional information not in the ms, or briefly explain why the item is not applicable/relevant for your study

"The generalizability is likely limited to people with harmful alcohol use or alcohol dependence, seeking help for their drinking on the internet."

"There were no differences in alcohol related problems (AUDIT) or dependence criteria (ICD-10) between those who accepted and those who declined participation. The high attrition rate also limits the generalizability of our results."

21-ii) Discuss if there were elements in the RCT that would be different in a routine application setting

Discuss if there were elements in the RCT that would be different in a routine application setting (e.g., prompts/reminders, more human involvement, training sessions or other co-interventions) and what impact the omission of these elements could have on use, adoption, or outcomes if the intervention is applied outside of a RCT setting.

	1	2	3	4	5	
subitem not at all important	○	○	○	◉	○	essential

Rensa markering

Ditt svar är för stort. Testa att förkorta en del av dina svar.

Does your paper address subitem 21-ii?

Copy and paste relevant sections from the manuscript (include quotes in quotation marks "like this" to indicate direct quotes from your manuscript), or elaborate on this item by providing additional information not in the ms, or briefly explain why the item is not applicable/relevant for your study

"In the current study we aimed to come as close as possible to the intended target group of the intervention when used in regular service at alkoholhjälpen.se. The trial was conducted in the same setting. Information on needed language skills and the limitations of the interventions in helping those with severe psychiatric problems were provided, but no other criteria was used to exclude participant that fulfilled the criteria set for harmful use or alcohol dependence."

OTHER INFORMATION

23) Registration number and name of trial registry

Does your paper address CONSORT subitem 23? *

Copy and paste relevant sections from the manuscript (include quotes in quotation marks "like this" to indicate direct quotes from your manuscript), or elaborate on this item by providing additional information not in the ms, or briefly explain why the item is not applicable/relevant for your study

"Trial registration: ClinicalTrials.gov, identifier: NCT02377726, https://clinicaltrials.gov/ct2/show/NCT02377726"

24) Where the full trial protocol can be accessed, if available

Ditt svar är för stort. Testa att förkorta en del av dina svar.

Does your paper address CONSORT subitem 24? *

Cite a Multimedia Appendix, other reference, or copy and paste relevant sections from the manuscript (include quotes in quotation marks "like this" to indicate direct quotes from your manuscript), or elaborate on this item by providing additional information not in the ms, or briefly explain why the item is not applicable/relevant for your study

Translated original protocol is provided as appendix.

25) Sources of funding and other support (such as supply of drugs), role of funders

Does your paper address CONSORT subitem 25? *

Copy and paste relevant sections from the manuscript (include quotes in quotation marks "like this" to indicate direct quotes from your manuscript), or elaborate on this item by providing additional information not in the ms, or briefly explain why the item is not applicable/relevant for your study

Grants:
Sven Andreasson, Swedish Research Council https://forte.se/ Grant nr 2013-1798
Magnus Johansson, Doctoral School in Health Care Sciences, https://ki.se/en/nvs/doctoral-projects

The funders had no role in study design, data collection and analysis, decision to publish, or preparation of the manuscript.

X27) Conflicts of Interest (not a CONSORT item)

Ditt svar är för stort. Testa att förkorta en del av dina svar.

X27-i) State the relation of the study team towards the system being evaluated

In addition to the usual declaration of interests (financial or otherwise), also state the relation of the study team towards the system being evaluated, i.e., state if the authors/evaluators are distinct from or identical with the developers/sponsors of the intervention.

	1	2	3	4	5	
subitem not at all important	○	○	◉	○	○	essential

Rensa markering

Does your paper address subitem X27-i?

Copy and paste relevant sections from the manuscript (include quotes in quotation marks "like this" to indicate direct quotes from your manuscript), or elaborate on this item by providing additional information not in the ms, or briefly explain why the item is not applicable/relevant for your study

The first author (MJ) has configured the internet platform and co-authored the program used in the study. The program is licensed under creative commons and the internet platform Drupal is open-source. MJ, PL and MG are employed at the Addiction eClinic, both part of the Stockholm Center of Dependency disorders that offers face-to-face and internet-based treatments for alcohol use disorders, including alkoholhjalpen.se.

About the CONSORT EHEALTH checklist

As a result of using this checklist, did you make changes in your manuscript? *

- ○ yes, major changes
- ◉ yes, minor changes
- ○ no

Ditt svar är för stort. Testa att förkorta en del av dina svar.

What were the most important changes you made as a result of using this checklist?

Ditt svar

How much time did you spend on going through the checklist INCLUDING making changes in your manuscript *

About five to six hours of active work

As a result of using this checklist, do you think your manuscript has improved? *

- (x) yes
- ( ) no
- ( ) Övrigt:

Would you like to become involved in the CONSORT EHEALTH group?

This would involve for example becoming involved in participating in a workshop and writing an "Explanation and Elaboration" document

- ( ) yes
- (x) no
- ( ) Övrigt:

Rensa markering

Ditt svar är för stort. Testa att förkorta en del av dina svar.

Any other comments or questions on CONSORT EHEALTH

Ditt svar

STOP - Save this form as PDF before you click submit

To generate a record that you filled in this form, we recommend to generate a PDF of this page (on a Mac, simply select "print" and then select "print as PDF") before you submit it.

When you submit your (revised) paper to JMIR, please upload the PDF as supplementary file.

Don't worry if some text in the textboxes is cut off, as we still have the complete information in our database. Thank you!

Final step: Click submit !

Click submit so we have your answers in our database!

Skicka

Skicka aldrig lösenord med Google Formulär

Det här innehållet har varken skapats eller godkänts av Google. Anmäl otillåten användning - Användarvillkor - Sekretesspolicy

Google Formulär

Ditt svar är för stort. Testa att förkorta en del av dina svar.
